# Cross-talk of the biotrophic pathogen *Claviceps purpurea* and its host *Secale cereale*

**DOI:** 10.1186/s12864-017-3619-4

**Published:** 2017-04-04

**Authors:** Birgitt Oeser, Sabine Kind, Selma Schurack, Thomas Schmutzer, Paul Tudzynski, Janine Hinsch

**Affiliations:** 1 Institut für Biologie und Biotechnologie der Pflanzen, Westfälische Wilhelms-Universität, D-48143 Münster, Germany; 2 Leibniz Institute of Plant Genetics and Crop Plant Research (IPK), Gatersleben, Germany

**Keywords:** Host-pathogen interaction, Effectors, *Claviceps purpurea*, Biotrophic pathogen, Transcriptome

## Abstract

**Background:**

The economically important Ergot fungus *Claviceps purpurea* is an interesting biotrophic model system because of its strict organ specificity (grass ovaries) and the lack of any detectable plant defense reactions. Though several virulence factors were identified, the exact infection mechanisms are unknown, e.g. how the fungus masks its attack and if the host detects the infection at all.

**Results:**

We present a first dual transcriptome analysis using an RNA-Seq approach. We studied both, fungal and plant gene expression in young ovaries infected by the wild-type and two virulence-attenuated mutants. We can show that the plant recognizes the fungus, since defense related genes are upregulated, especially several phytohormone genes. We present a survey of *in planta* expressed fungal genes, among them several confirmed virulence genes. Interestingly, the set of most highly expressed genes includes a high proportion of genes encoding putative effectors, small secreted proteins which might be involved in masking the fungal attack or interfering with host defense reactions. As known from several other phytopathogens, the *C. purpurea* genome contains more than 400 of such genes, many of them clustered and probably highly redundant. Since the lack of effective defense reactions in spite of recognition of the fungus could very well be achieved by effectors, we started a functional analysis of some of the most highly expressed candidates. However, the redundancy of the system made the identification of a drastic effect of a single gene most unlikely. We can show that at least one candidate accumulates in the plant apoplast. Deletion of some candidates led to a reduced virulence of *C. purpurea* on rye, indicating a role of the respective proteins during the infection process.

**Conclusions:**

We show for the first time that- despite the absence of effective plant defense reactions- the biotrophic pathogen *C. purpurea* is detected by its host. This points to a role of effectors in modulation of the effective plant response. Indeed, several putative effector genes are among the highest expressed genes *in planta*.

**Electronic supplementary material:**

The online version of this article (doi:10.1186/s12864-017-3619-4) contains supplementary material, which is available to authorized users.

## Background

In nature plants are exposed to a vast number of potential pathogens. To protect themselves from infections they rely on their innate immune system which mainly consists of two lines of defense [1]. The first line, or basal defense, is pathogen unspecific as broadly conserved pathogen associated molecular patterns (PAMPs) are recognized via transmembrane pattern recognition receptors (PRR) [2, 3]. This leads to PAMP triggered immunity (PTI) of the plant. However, pathogens are able to bypass this mechanism by the secretion of so called “effector proteins”. These proteins impede PTI by e.g. suppressing defense mechanisms, causing a susceptible interaction (ETS = effector triggered susceptibility). The next step in the evolutionary arms race of host and pathogen is the recognition of (avirulence) effector proteins by plant disease resistance (R) proteins [1, 4]. This second line of defense results in an induction of strong defense mechanisms, often including a hypersensitive resistance response (HR) at the infection site (ETI = effector triggered immunity) which especially hinders the spreading of biotrophic and hemibiotrophic pathogens. In turn, natural selection leads to the avoidance of ETI either by losing/altering the avirulence effector or by gaining additional effector(s). Thus, in plant-pathogen interactions a complex network of effectors and R-proteins has evolved [1].

In recent years major progress has been made in the understanding of the function of effector proteins in plant-fungus interactions. Especially their role in biotrophic and hemibiotrophic fungi has been under intensive investigation as these lifestyles require intact, living plant cells. Generally, effectors can modulate the host’s metabolism and directly influence defense reactions thus helping with establishing compatible interactions [5–7]. In addition to these proteinaceous effectors, secreted fungal low-molecular weight compounds like reactive oxygen species [8] or secondary metabolites, like plant hormones [9] have turned out to be important for compatible interactions.

The highly specialized interaction of the Ergot fungus *Claviceps purpurea* and rye is exceptional because no obviously visible signs of plant defense reactions occur in infected plant tissue. In contrast, the fungus manages to keep plant cells alive for a prolonged time period and is therefore classified to be a true biotroph [10]. It is most likely that it directly influences both, the metabolism as well as the defense machinery of the host plant by secreted effectors, however experimental proof is missing. Characteristically, the infection process of *C. purpurea* is organ specific, as solely flowers of blooming grass ears are infected. The fungus penetrates the stigmatic hairs and grows towards the transmitting tissue of the ovary. During this phase, *C. purpurea* grows mainly intercellularly but invasive hyphae which are completely covered by the host plasma membrane have also been documented [11]. Thus, a secretion of effectors into the plant apoplast as well as the translocation into the host cell might be possible in the *C. purpurea*-rye interaction. Once the fungus reaches the base of the ovary, it taps into the vascular system of the plant to gain nutrients and progressively replaces the plant tissue with fungal material. Finally, instead of a caryopsis an ergot alkaloid containing sclerotium is formed serving as overwintering and sexual reproduction structure.

A concerted cytological and molecular genetic approach has led to the identification of several virulence factors (see recent review [12]). Apart from pectin-degrading enzymes, several signaling components like MAP kinases [13–15], ROS generating NADPH oxidases [16], and recently cytokinins produced by the fungus have been shown to be essential for successful infection [17, 18]. In addition, two factors turned out to be of special interest for the *C. purpurea-*rye interaction. These are the transcription factor Cptf1 (a homolog of the yeast Ap1) and the small GTPase Cpcdc42. Deletion of these genes did not only reduce the virulence of the fungus but, even more interesting, both deletion mutants triggered massive ROS production *in planta*, indicating a plant defense response [19, 20]. Therefore, we included these mutants in the first genome-wide *in planta* expression study of this interaction system reported here. Since genomes of both partners are available [21, 22], we used a dual RNA-Seq approach which allows the identification of transcriptomic changes of both partners [23, 24]. We demonstrate that the transcriptome of the host is differentially affected during the infection with the wild-type strain of *C. purpurea* and the two virulence-attenuated mutants. Strikingly, apart from several potential virulence factors, a broad set of putative effector genes is upregulated *in planta*, some of them obviously controlled by Cptf1. Thus, functional analyses of selected effector candidates were performed.

## Results

### Experimental design and data evaluation

**Fig. 1:**
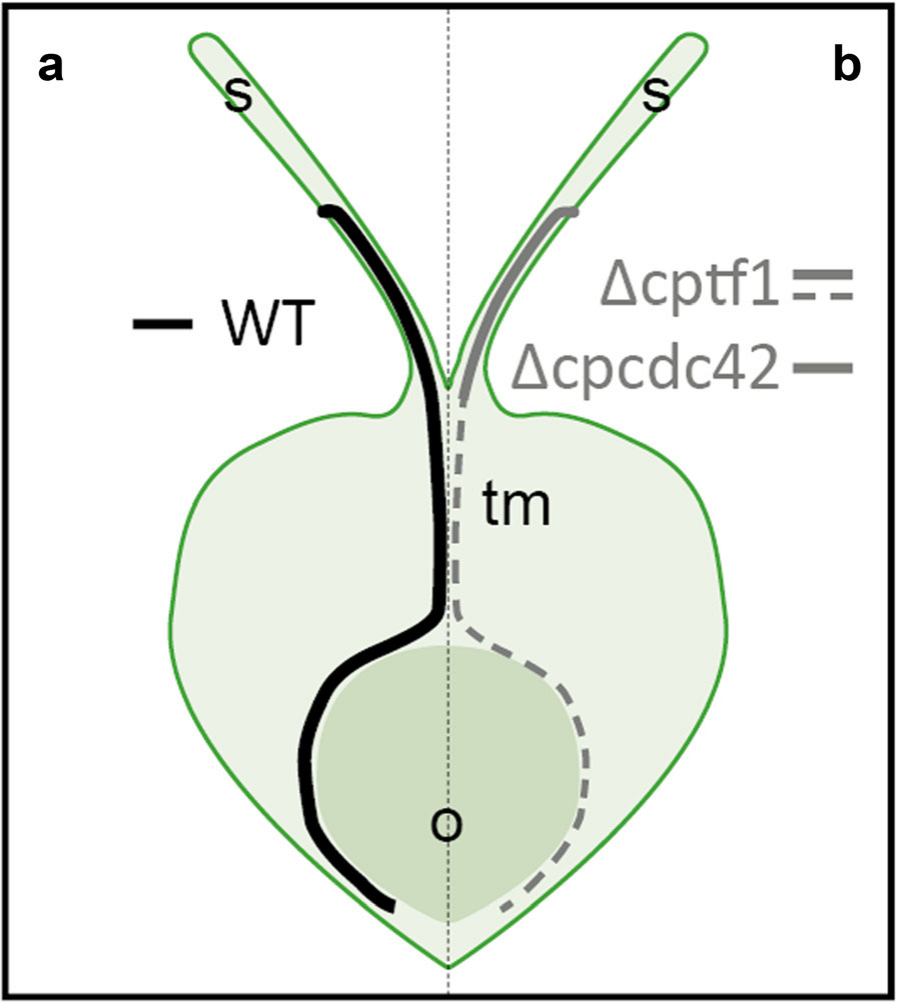
Infection stages of *C. purpurea* strains at the time point of sample collection. **a** The wild-type *Cp*20.1 (*black line*) enters the ovary at the stigmatic hairs (s), passes the transmitting tissue (tm), grows around the ovule (o) and almost reaches the base of the ovary. **b** The virulence-attenuated mutant Δcptf1 (*grey continuous* and *dashed line*) also penetrates the stigmatic hairs but reaches the base only sparsely. Δcpcdc42 (*grey continuous line*) penetrates the stigmatic hairs but does not enter the ovarian cap

To study the communication network between *Claviceps purpurea* and its major host plant *Secale cereale* (rye), we isolated RNA from about 90–120 ovaries each from plants infected with water (mock control), the wild-type *Cp*20.1 and two mutants (∆cpcdc42 and ∆cptf1). With these mixed transcriptome samples we performed Illumina RNA Sequencing with a paired-end protocol. Due to the large host genome size (7.9 Gb) [21] and the extremely low content of fungal material in the samples, we limited the sample number to one sample per treatment and performed a very deep sequencing (10^8^ reads per sample) of one time point (5 days post inoculation). To verify the data, qRT-PCR of candidate genes of two additional replicates were performed as a second step. The gene expression profiles obtained in this study would be originated largely from wild-type infected ovaries, in which the fungus reached the base of the ovary, while the infection process of the two mutants attenuates in the stigmatic hairs or comes to a complete stop in the transmitting tissue (Fig. 1).

**Fig. 2:**
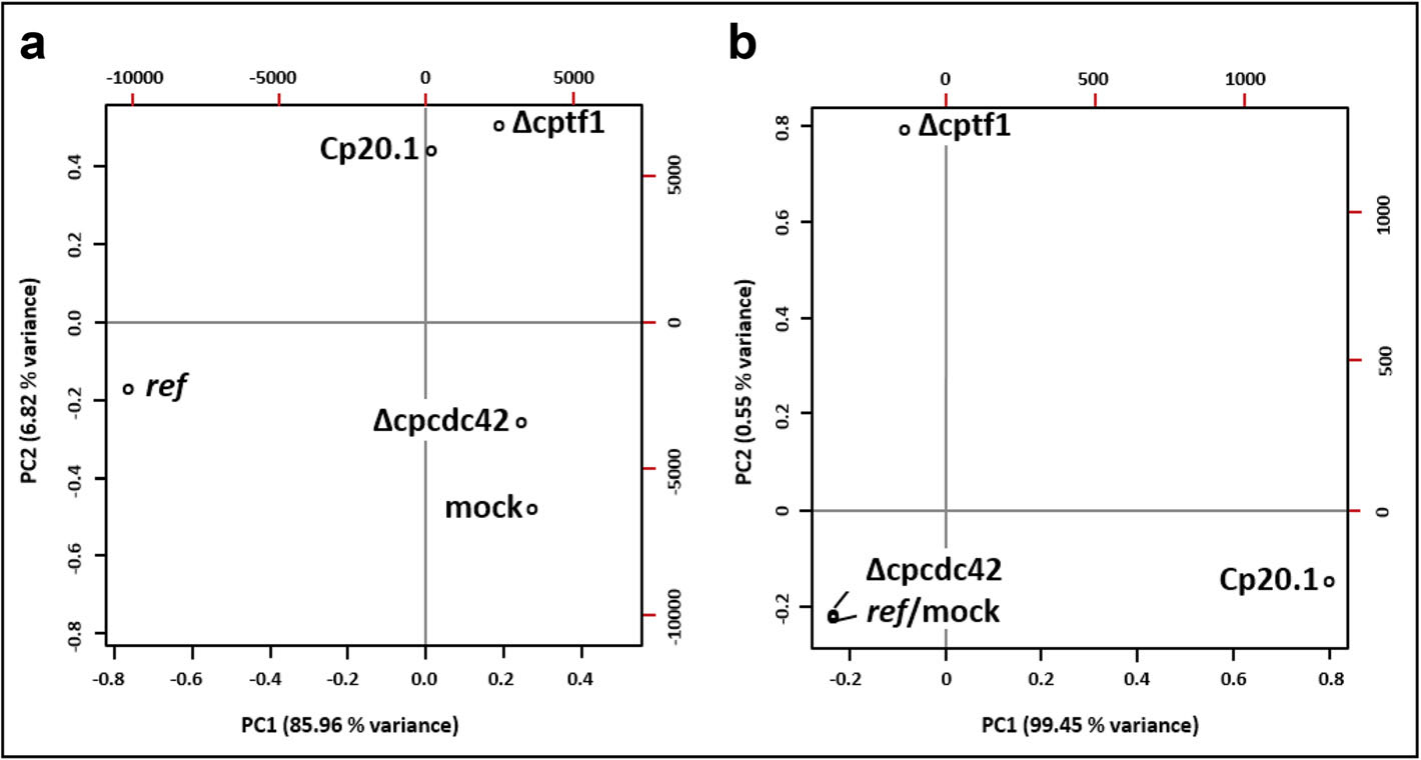
Principal component analysis of *de novo* assembled transcripts: **a** plant sequences and **b** fungal sequences. Compared were the FPKM values of the reads matching the transcripts of all four samples (mock, *Cp*20.1 infected, ∆cptf1 infected, ∆cpcdc42 infected) plus a reference data set for plant and fungal sequences (*ref*; all data points set to zero)

After processing of the reads, we obtained between 25 and 28x10^6^ paired-end reads for the different samples (Additional file 1). These were then assembled *de novo* and sorted into “plant-“(80.4%) and “fungal”-derived (15.2%) via BLAST analyses against the *C. purpurea* genome, a rye cDNA database [25] and NCBI databases. Transcripts mapping to both, plant and fungal sequences, were excluded (4.5%). A principal component analyses (PCA) of these data sets shows that the four plant transcriptomes were all significantly different, indicating that the infection process of the strains induces specific responses within the host (Fig. 2a). The sizes of the fungal data sets differ considerably with 9.81% (*Cp*20.1), 1.62% (Δcptf1) and 0.03% (Δcpcdc42) of reads mapping to the *C. purpurea* reference (Additional file 1) and obviously reflect the colonization levels of the strains. The PCA also reveals significantly different transcriptomes for the three fungal data sets (Fig. 2b). However, due to the low quantity of fungal transcripts in the ∆cpcdc42 infected material, this data set was not included in further quantitative evaluations. The reads of the other data sets were mapped against the *C. purpurea* and the rye genome to analyze the transcriptomic changes during infection in more detail [21, 22].

### Transcriptomic changes in host plants after fungal infection

**Fig. 3:**
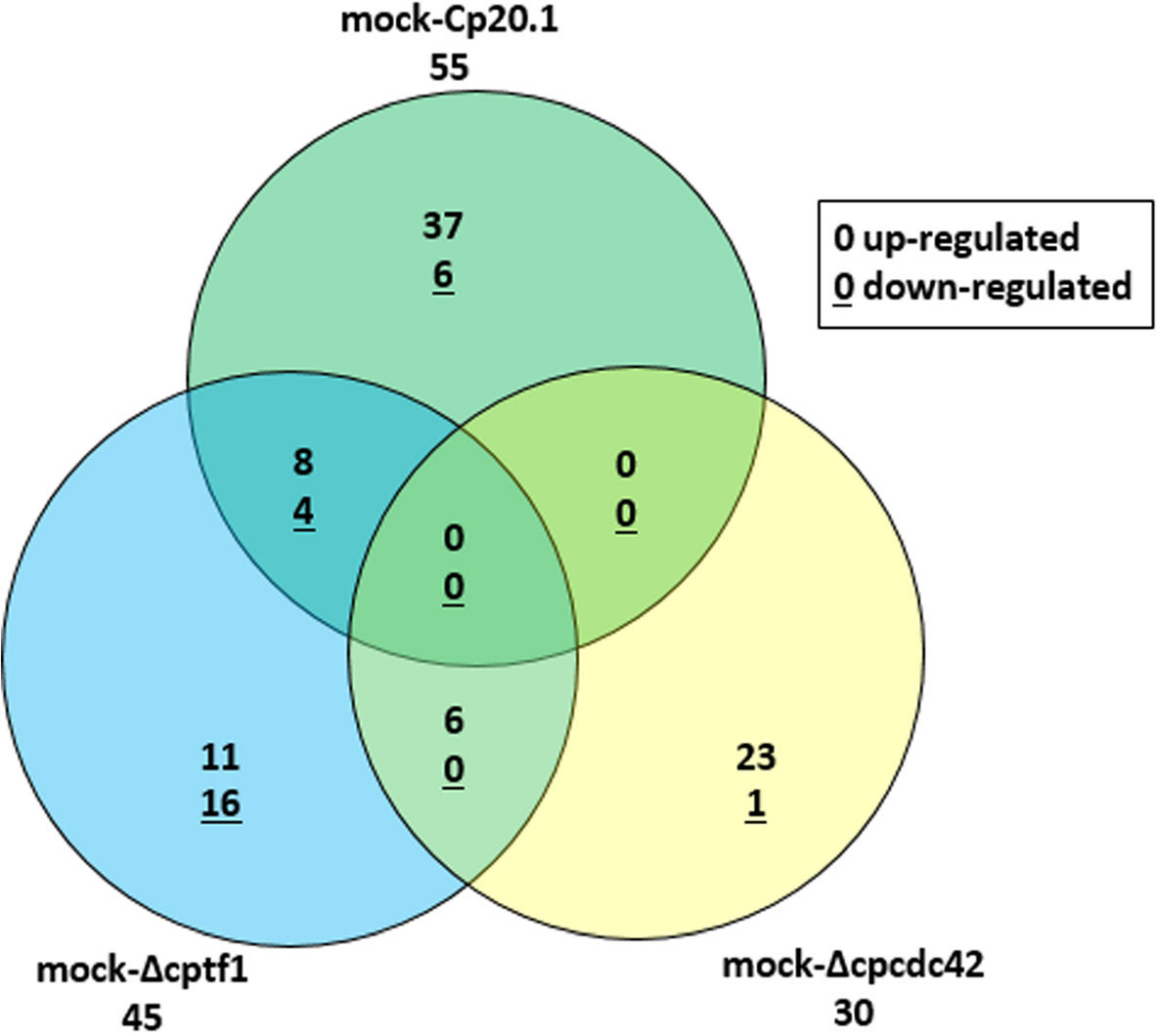
Venn diagram of differentially expressed plant genes. Overlapping areas show the numbers of genes found to be congruently regulated comparing mock-*Cp*20.1 (*green*,45 *up*, 10 *down*), mock-Δcptf1 (*blue*, 19 *up*, 20 *down*), and mock-Δcpcdc42 (*yellow*, 23 *up*, 7 *down*), resp

A comparative analysis using the conservative dispersion method “blind” yields a set of 112 candidate genes which show (within the limits of this experimental approach) significantly different expression levels between the four rye data sets (Fig. 3; Additional file 2). Compared to the mock-treated control, infection with *Cp*20.1 causes a significant change in gene expression (45 up regulated, 10 down) in rye ovaries. Among the up-regulated genes, several encode potential pathogenesis-related proteins like for example a β-1,3 glucanase and a peroxidase. Two homologs of the disease resistance protein Rg4 show significantly altered expression only in *Cp*20.1 infected material. While expression of Sc3Loc00972435.4 is slightly induced during infection, expression of Sc3Loc01905034.2 is repressed more drastically. Interestingly, the two data sets of the virulence-attenuated strains reveal differential expression of other defense genes. For example, both mutants induce the expression of a chitinase (Sc2Loc00083431.2). Additionally, induction of further plant defense genes specifically induced by Δcptf1 or Δcpcdc42 can be observed. In Δcptf1 a gene encoding an enzyme involved in the biosynthesis of hydroxamic acids in rye is induced. These compounds have been shown to have anti-fungal activity and to play an important role in disease resistance [26, 27]. Furthermore, the expression of a gene with high homology to an inhibitor for fungal xylanases (Sc4Loc00580338.2) as well as a predicted disease resistance protein (Sc4Loc01458017.2) is significantly induced solely in Δcptf1 infected ovaries. Infection with Δcpcdc42 specifically induces gene expression of, amongst others, a glutathione S-transferase (GST) (Sc6Loc01250185.2), a P450 monooxygenase (Sc3Loc01925247.1) and a reticuline oxidase-like protein (Sc1Loc01953392.2).

Noticeably, the expressions of genes which are associated with the plant hormone auxin are significantly induced in all data sets compared to the control. Besides the induction of expression of an auxin responsive gene (Sc1Loc01149658.2) in *Cp*20.1 infections, two indole-3-acetic acid-amido synthetase-like genes (Sc3Loc01478542.1, Sc2Loc00096015.6) are upregulated in both, *Cp*20.1 and Δcptf1 infected ovaries. This induction is not present in Δcpcdc42 infections but here, four homologs of the auxin inducible gene 5NG4 (Sc1Loc01436284.1, Sc2Loc00251684.1, Sc6Loc00348292.4, Sc2Loc01270200.1) are significantly upregulated. Moreover, a gene encoding a cytokinin oxidase/dehydrogenase (*scckx1*, Sc3Loc01734780.3) and a jasmonate induced gene (Sc6Loc01509737.4) are specifically upregulated exclusively in this interaction.

### Fungal genes expressed during infection

To allow a broad and unbiased evaluation of the fungal transcriptome during infection, first, all genes identified in the *Cp*20.1 genome (8846) were grouped into 4 categories according to their expression levels: basic expression (0–100 FPKM), low expression (>100-200), medium expression (>200-640) and high expression (>640). Next the distribution of GO terms was determined within categories low, medium and high (Additional file 3). A detailed list of the category “biological processes” is given in Additional file 4 and additionally this group was visualized in a REVIGO scatterplot (Additional file 5). The analysis shows that a majority of expressed genes encodes proteins involved in “metabolism”, “cell organization and biogenesis” as well as in “generation of precursor metabolites and energy”, “reproduction”, “cell differentiation” and “development” during this early phase of infection.

**Fig. 4:**
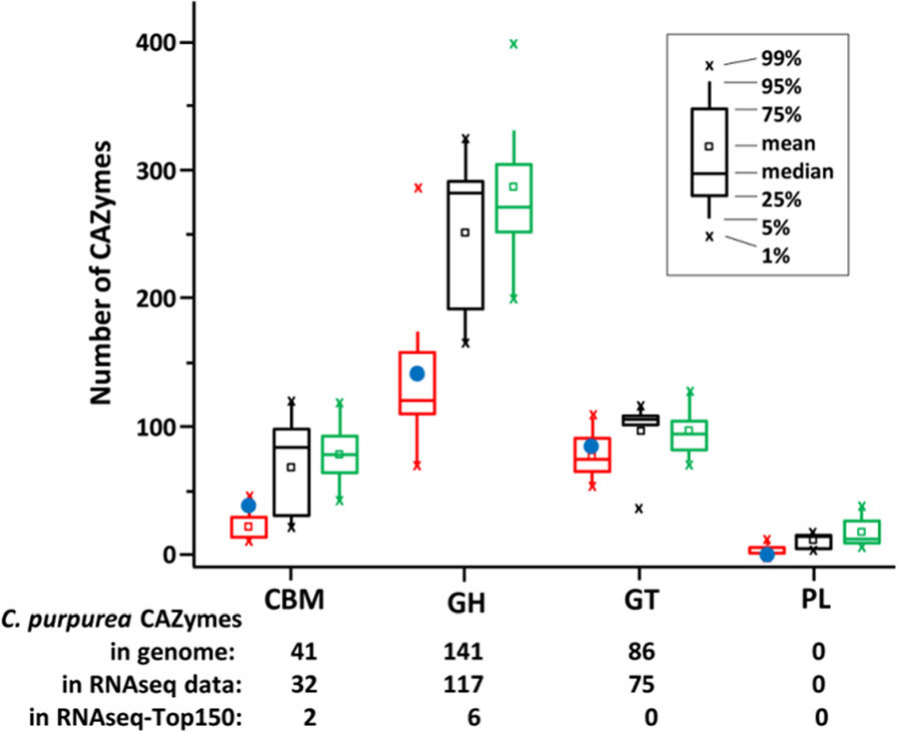
*C. purpurea* CAZymes in comparison to CAZymes of other plant pathogenic fungi. *C. purpurea* data points (*blue dots*) were integrated in a plot showing the number of CAZymes (GH = glycosyl hydrolases, GT = glycosyl transferases, PL = polysaccaride lyases, CBMs = carbohydrate binding molecules) for biotrophic (*red*), hemibiotrophic (*black*) and necrotrophic (*green*) pathogens (modified after [28])

In total, expression of 6859 fungal genes could be observed in wild-type infected ovaries. As expected, among the 150 most highly expressed genes (ribosomal proteins excluded, Additional file 6) the majority (~90%) encodes for proteins involved in growth and development or for components of the primary metabolism. Typical for plant-infecting fungi, *C. purpurea* is well equipped with “carbohydrate-active enzymes” (CAZymes) including plant cell wall degrading as well as nutritional enzymes, and most (~80%) of these are mapped by RNA-Seq reads ([28], Fig. 4). In total, 10 CAZyme genes can be found in the 150 most highly expressed genes (Additional file 6). A comparative analysis of fungi with different life styles classifies this proportion as a moderate expression level, substantiating the biotrophic nature of the interaction of *C. purpurea* and rye (Fig. 4).

Among the highly expressed genes (Additional file 6), *cppg1* (*cp6977*)*,* a gene encoding a polygalacturonase which has already been shown to be an important virulence factor in the *C. purpurea* – rye interaction, was detected [13]. Further already analyzed genes included in this list are the MAPkinase encoding gene *cpmk1* (*cp1700*) which has also been shown to be a virulence factor for *C. purpurea* [14], as well as *cpsod1*, encoding a superoxide dismutase (*cp7438*; [29]) which is not essential for infection. In addition, several potential effector-encoding genes are included (see below).

A comparison of the *Cp*20.1 data with the data set obtained for the virulence attenuated mutant ∆cptf1 shows, despite a clear reduction of reads due to reduced fungal biomass in the samples (see above), a still rather broad distribution of GO terms (data not shown). A cautious comparative analysis of these two data sets yields a list of 48 candidate genes which show significantly different expression levels within the limits of this experimental approach (no expression in the ∆cptf1 mutant; Additional file 7). For instance, genes encoding a sugar transporter (*cp2739*), a cellulose degrading enzyme (*cp4457*), a β-1,3-glucanase (*cp3134*) and several putative effectors (see below) are downregulated in ∆cptf1 during infection.

### Data validation by qRT-PCR

**Fig. 5:**
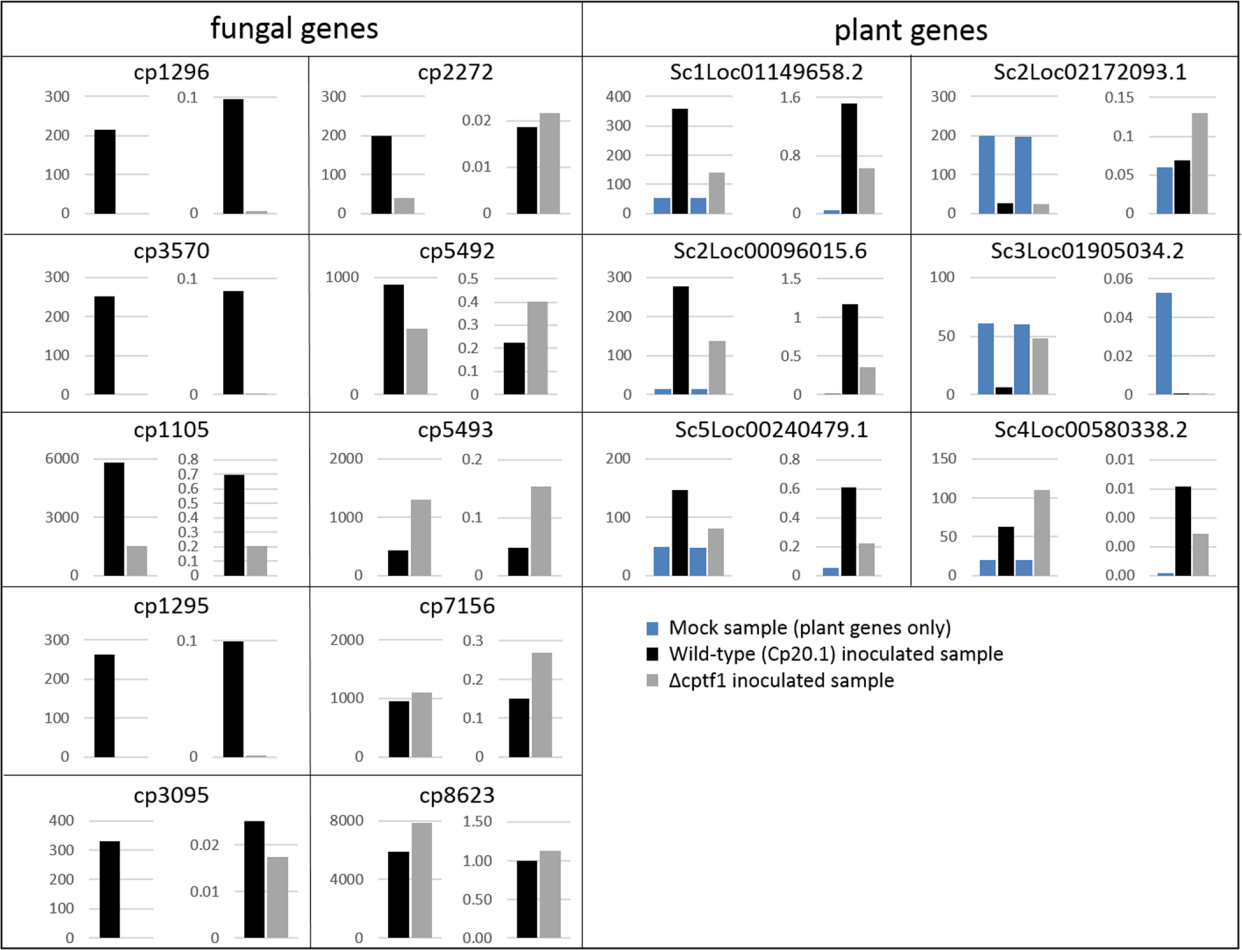
Validation of RNA-Seq data by qRT-PCR: Relative mRNA levels are comparable to RNA-Seq data in most cases. Comparison of FPKM values (*left* figure, RNA-Seq data) and relative expression (normalized to reference genes, *right* figure, qRT-PCR results) of various genes in the 2nd biological replicate. Shown are representative genes that were identified as induced during the infection or down-regulated in Δcptf1. *cp1296*: invertase; *cp3570*: xylanase; *cp1105*, *cp1295, cp3095*: effector candidate; *cp2272*: sge1-homologue; *cp5492*, *cp5493*, *cp7156*, *cp8623*: effector candidate; Sc1Loc01149658.2, Sc2Loc00096015.6: auxin-responsive genes; Sc5Loc00240479.1: chitinase; Sc2Loc02172093.1: flower development Sc3Loc01905034.2: plant defense; Sc4Loc00580338.2: xylanase inhibitor

To validate the results obtained in the RNA-Seq approach, the expression levels of several plant and fungal candidate genes were measured in two biological repeats (Fig. 5). The candidate genes were chosen because their predicted functions are of interest from published (*cp3570*: xylanase, Sc4Loc00580338.2: xylanase inhibitor, [30]) or current research topics (Sc1Loc01149658.2, Sc2Loc00096015.6: auxin-responsive genes; Sc5Loc00240479.1: chitinase*,* Sc2Loc02172093.1: flower development; Sc3Loc01905034.2: plant defense, *cp1296:* invertase; *cp2272*: *sge1*-homologue; and several potential effectors). The relative expression of five plant genes (Sc1Loc01149658.2, Sc2Loc00096015.6, Sc5Loc00240479.1, Sc2Loc02172093.1, Sc3Loc01905034.2) which were found significantly differentially expressed in the *Cp*20.1 infected material were tested with quantitative qRT-PCR and four could be confirmed (all except Sc2Loc02172093.1). Two genes (Sc2Loc00096015.6 and Sc4Loc00580338.2) are induced in plants inoculated with Δcptf1, both also showed increased relative expression levels when tested with qRT-PCR. For *C. purpurea* the expression patterns of *cp1105, cp1295, cp1296, cp2272, cp3095, cp3570, cp5492, cp5493, cp7156* and *cp8623* were analyzed. Compared to the expression of housekeeping genes, *cp1105, cp5493, cp7156* and *cp8623* are highly expressed during infection as predicted by the RNA-Seq data set. The genes *cp1295, cp3095, cp1296* and *cp3570* were significantly differentially regulated in the wild-type and Δcptf1 and indeed, this could be validated by qRT-PCR for *cp3095* and *cp3570*, for *cp1295* and *cp1296* one out of two biological replicates was differing. The different expression levels of *cp2272* and *cp5492* were not considered as significant according to the RNA-Seq data, and the pattern could actually not be confirmed by qRT-PCR. Overall, our analyses show that the conservative methods applied to the RNA-Seq data yielded a small but reliable data set.

### Functional analysis of potential effectors

Besides the general overview of *C. purpurea* and rye transcriptomes during the interaction, one particular objective of this study was the identification of potential fungal effectors, as these proteins have been shown to be of major importance in plant-pathogen interactions [6]. However, the identification of effector proteins in filamentous fungi has turned out to be difficult. In contrast to oomycete effectors which share common N-terminal amino acid sequence motifs like the most prominent “RxLR” or the “Crinkler” motif [31], no such conserved motif has been identified in filamentous fungi. However, several common criteria have been attributed to so far identified effectors [7]. Accordingly, a genome wide bioinformatic analysis was conducted applying these criteria. Firstly, the *C. purpurea* genome was screened for predicted proteins with a size of 10–330 amino acids. Out of these candidates only those containing a signal peptide (SP; predicted by programs SignalP, SigCleave, Phobius and TargetP1.1) were further investigated. In the next steps, proteins containing a transmembrane domain, a GPI anchor or an endoplasmic reticulum (ER) retention signal were excluded (using Phobius/TMHMM/DAS, fragAnchor, ScanProsite). In total, 470 predicted proteins fulfilled all criteria and therefore represent effector protein candidates within the *C. purpurea* genome (Additional file 8). As shown in other fungi, many of these potential effector genes are clustered, often showing very high homology, i.e. they probably are the result of recent duplication events: 176 effector genes are localized in 67 clusters, most of them with 2–3 genes, up to 6 cluster genes (Additional file 8).

As a high expression rate during infection is another typical feature of effectors, the effector candidates were compared to the results of the RNA-Seq analysis. Strikingly, 25 candidates were included in the 150 most highly expressed genes of *Cp*20.1 during infection and five even belonged to the top ten highly expressed genes, e.g. *cp8623* (2nd), *cp1105* (3rd) (Additional file 6). Several of the clustered effector genes also are included in the top 150 list, e.g. *cp5492* **.** Additionally, seven potential effector candidate genes are downregulated in the virulence attenuated ∆cptf1 mutant compared to the wild-type *Cp*20.1, e.g. *cp3095* (Additional file 7). The large number of potential effector genes, especially the high level of duplications point to a high redundancy of the system, making a specific dramatic effect of the loss of a single gene unlikely. The low homologous recombination rate of the fungus (1%) does not allow a systematic deletion approach but we initiated a functional analysis of some of the special effector candidates highlighted above, to get an idea of the role of these proteins in this highly specialized interaction system.

*cp1105* encodes a 75 aa polypeptide, with a predicted SP of 23 aa. The processed protein sequence contains 7 cysteine (Cys) residues, making up 13%. No paralogs occur in the *C. purpurea* genome and no orthologs could be detected in other organisms.

The product of *cp5492* has a predicted amino acid sequence of 98 aa. The processed protein contains 6 Cys residues accounting for 8% of the aa sequence. The encoded protein has low similarity to other *C. purpurea* proteins (Cp7654 E = 5.9e-15; Cp5497 E = 3.1e-11; Cp1691 E = 9.5e-9; Cp1529 E = 7.2e-8; Cp5493 E = 5.6e-7). Noticeably, *cp5493* (37% identity on aa level) is located adjacent to *cp5492* and also encodes a potentially secreted 98 aa protein (8% Cys residues) classified as potential effector. For Cp5492 and Cp5493, no orthologs could be identified in other organisms.

*cp3095* lies within a cluster of five similar potential effector genes, designated effector cluster 10 (EfCl10). The putative effector genes in this cluster are *cp3095, cp3096, cp3098, cp3099* and *cp3100. cp3097* is not an effector candidate : BLAST analysis revealed similarity to a transposase-like protein of the fungus *Pochonia clamydospora.* Similarities between the putative effectors range from 54 to 90%.

The predicted gene product of *cp3095* consists of 94 amino acids with 4.3% cysteine; the predicted gene product of *cp3096* has 114 amino acids and contains 5.3% cysteine. 86% of the amino acid sequence is identical.

Gene *cp8623* encodes 90 aa with 3% Cys. Besides the predicted SP, the protein contains a domain with similarity to lysM domains (IPR018392; NCBI CDD E = 3.53e-04). It shows low similarity to Cp2126 (E = 3.5e-9) which is mainly based on the lysM-like domain. BLAST analyses also revealed low similarity of Cp8623 to an uncharacterized protein of *Colletotrichum fioriniae* (XP_007590355.1, E = 5e-20) and a putative m23b peptidase of *C. gloeosporioides* Nara gc5 (XP_007275188.1; E = 4e-16) which is mainly based on the presence of similar lysM-like domains as well.

**Fig. 6:**
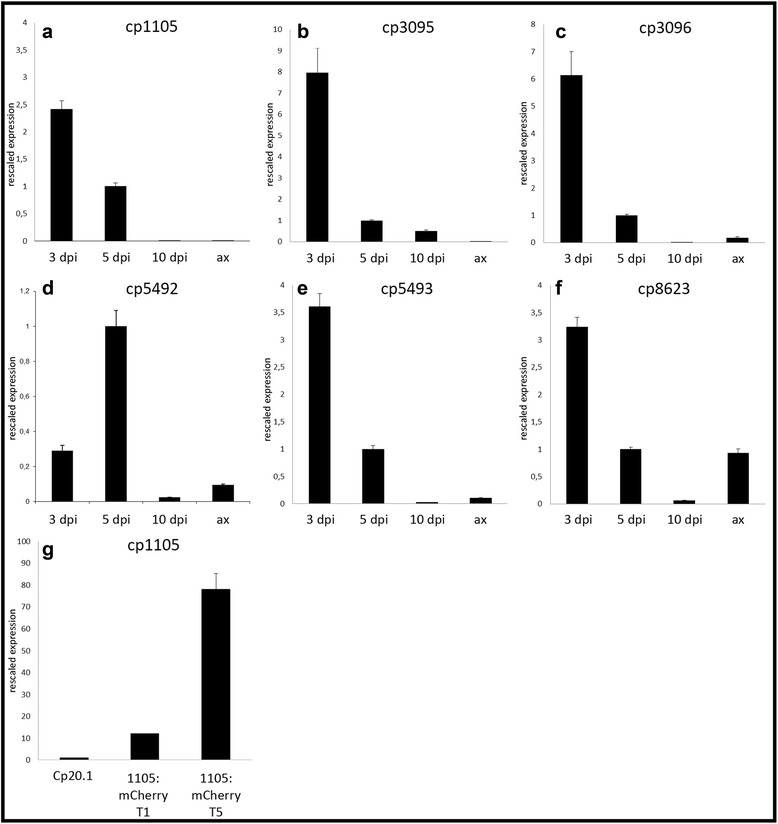
Expression of effector candidates. Expression levels of *cp1105* (**a** + **g**)*, cp3095* (**b**)*, cp3096* (**c**), *cp5492* (**d**)*, cp5493* (**e**) and *cp8623* (**f**) in the wild type *Cp*20.1 during infection 3, 5 and 10 dpi as well as in axenic culture was determined by qRT-PCR and normalized to housekeeping genes. Expression of *cp1105* (**a**)*, cp3095* (**b**)*, cp3096* (**c**), *cp5492* (**d**)*, cp5493* (**e**) was strongly induced 3 and 5dpi and decreased 10 dpi as well as in axenic cultures. No clear expression pattern was detectable for *cp8623* (**f**). Here, comparable expression rates were observed 5dpi *in planta* and in axenic culture. Expression of *cp1105* was also determined in strains harboring a cp1105:mCherry construct from axenic culture (**g**). The experiments were done in two independent biological replicates, exemplary results from the second replicate are shown. Error bars indicate standard deviation from technical replicates

The expression pattern of all candidate genes was verified by qRT-PCR with regard to the influence of Cptf (Fig. 5). Additionally, the expression pattern at different stages during wild-type infection and in liquid culture was analyzed (Fig. 6). Effectors are assumed to be of particular importance during the early infection phase before pathogens obtained access to nutrients and extensive growth. The expression of *cp1105*, *cp5492*, *cp5493, cp3095* and *cp3096* peaks in the very early and early infection stage (3 or 5 dpi) and is several fold decreased at 10 dpi compared to the peak. For *cp8623* no clear pattern was observed and expression levels were fluctuating between replicates. The expression of the other candidates in axenic culture was clearly below that of the early infection phase. Their expression pattern therefore supported their function as effector during *in planta* growth. Detailed functional characterizations using live cell imaging and deletion mutants were performed.

### Localization studies

Effector proteins of plant-pathogenic fungi have been shown to be either located within the host apoplast or to be translocated into the host cell. To identify the localization of the effector candidates, especially during the infection process on rye, *C. purpurea* strains expressing translational fusion proteins with mCherry proteins at the C-terminus of the effector candidates were produced. To preclude the possibility that no fluorescence could be observed for the other fusion constructs due to the extensive dilution of the proteins within the host cell, an enrichment strategy established by [32] was applied. The strategy relies on the addition of a nuclear localization signal (NLS) from simian virus large T-antigen [33] to the C-terminus of the fusion proteins. As the NLS is a universal signal, NLS containing proteins are transported to the nucleus. Proteins containing an N-terminal SP, like effector proteins, enter the secretion pathway before the C-terminal NLS redirects the protein. If the secreted protein is subsequently translocated into the host cytoplasm, it will artificially be redirected to the host nucleus. This in turn enables the detection of the fusion protein due to the artificial enrichment at this point. First, the triple NLS sequence was C-terminally added to the mCherry sequence in the plasmid pNDH-OCT (overexpression by oliC promotor [34]). Functionality of the construct was proven by transformation into *Cp*20.1 where it led to distinct fluorescence within the fungal nuclei (Additional file 9). All effector fusion constructs were under the control of the native promoters. The integration of the whole constructs was verified via PCRs (data not shown) and three independent transformants per construct were chosen for further investigations.

**Fig. 7:**
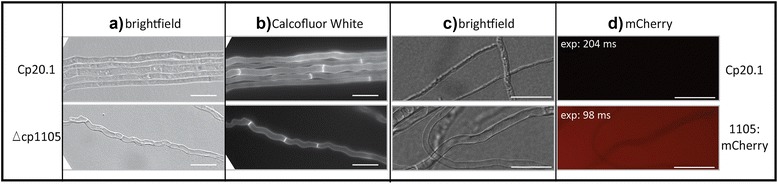
Microscopical analyses of Δcp1105 (**a** + **b**) and localization of Cp1105 reporter fusion protein (**c** + **d**) in axenic culture. Strains were cultivated on medium covered objective slides and incubated for 3 days before they were microscopically analyzed. **a** The hyphal morphology of Δcp1105 shows mainly undulated hyphae. **b** Calcofluor White Staining of septa. The mutants show a uniform septation pattern which is similar to the wild-type. **c** + **d** Fluorescence can be observed in the medium surrounding the hyphae. No significant fluorescence was observed within medium inoculated with the wild-type *Cp*20.1. exp: exposure time (Bars = 20 μm)

First, the strains were microscopically analyzed in axenic cultures. No fluorescence could be observed in the wild-type control. As expected due to the relatively low expression levels, no fluorescence could be observed for strains expressing *cp5492*:*mCherry*, *cp5493*:*mCherry* and *cp8623*:*mCherry*. Unexpectedly, two of three strains containing the *cp1105*:*mCherry* construct showed strong fluorescence in the media surrounding the hyphae (half of the exposure time of the wild-type control, Fig. 7). The intensity of the signal decreased more distantly from the hyphae and no fluorescence signal was detected within the fungal hyphae, indicating the secretion of Cp1105:mCherry. Because of the expected low expression of *cp1105* in axenic culture the expression levels were rechecked in the *mCherry* transformants and qRT-PCR results indeed revealed that the expression of *cp1105* was several-fold increased compared to *Cp*20.1 (Fig. 6g). These findings indicate that ectopic or multiple integrations of constructs influence the expression level of the native promoter.

To identify the localization pattern of the effector:mCherry fusion proteins *in planta*, all strains were used to inoculate rye ovaries. After verification that the strains were not impaired in virulence (honeydew and sclerotia formation) microscopical analyses were performed 3 and 5 dpi. No specific fluorescence could be observed for any of the mCherry strains nor for the wild-type control *Cp*20.1.

### Generation of deletion mutants

To characterize the function of the effector candidates during the infection of rye as well as in axenic cultures deletion mutants were generated via gene replacement strategies. PCR fragments (ca 1 kb) of the 5′ and 3′-flanking regions of the respective genes (*cp1105, cp8623*) were either fused to the phleomycin- or hygromycin-resistance cassette in the yeast-shuttle vector pRS426 (Additional file 10). The linearized constructs were then used to transform protoplasts of wild-type strain *Cp*20.1. The head-to-tail organization of *cp5492* and *cp5493* as well as of *cp3095* and *cp3096* allowed one-step double knockout strategies: For these approaches the hygromycin resistance gene was used and a marker split strategy was applied ([35]; Additional file 11).

Primary transformants were checked via diagnostic PCR analyses for the presence of correctly integrated replacement constructs. From 71 primary transformants for *cp1105* and 51 transformants for *cp8623*, transformants with correctly integrated replacement constructs were identified and single spore isolations lead to one homokaryotic deletion mutant each. Southern analyses confirmed the lack of the wild-type gene copy and a single integration event (Additional file 10). For *cp5492*/*cp5493* 45 transformants were analyzed and 3 independent double knockout mutants (Additional file 11) could be isolated. For *cp3095/cp3096* two double knockout mutants without additional ectopic integrations were identified from 40 primary transformants (Additional file 11). All knockout mutants were used for further characterization.

### Characterization of effector deletion mutants

To analyze if the deletion strains are impaired in axenic growth or virulence several assays were performed. The growth characteristics were assessed by plate assays and microscopic observation of hyphae growing on solid medium. To evaluate if the deletions affected the virulence, appearance of micro- and macroscopic infection symptoms was determined.

For the plate assay colony diameters were measured 12 dpi from the wild-type and the deletion strains of *cp1105* and *cp8623* growing on complete (BII) and minimal medium (Mantle). Indicated percentages refer to the colony diameter of *Cp*20.1 (Additional file 12). The measurements clearly show a reduced growth rate of Δcp1105 under the tested conditions. For Δcp8623 only a slightly reduced growth rate on standard Mantle medium was observed while it grows wild-type like on complete medium. Growth rates of the double deletion strains were not altered (data not shown). Microscopical analyses of ΔΔcp5492/cp5493, ΔΔcp3095/cp3096 and Δcp8623 hyphae did not reveal any morphological changes compared to *Cp*20.1 (data not shown). In contrast, hyphae of Δcp1105 were frequently undulated, a phenotype that is only rarely observed in the wild-type. Staining with Calcofluor White did not reveal any differences in septum formation in this mutant (Fig. 7).

**Fig. 8:**
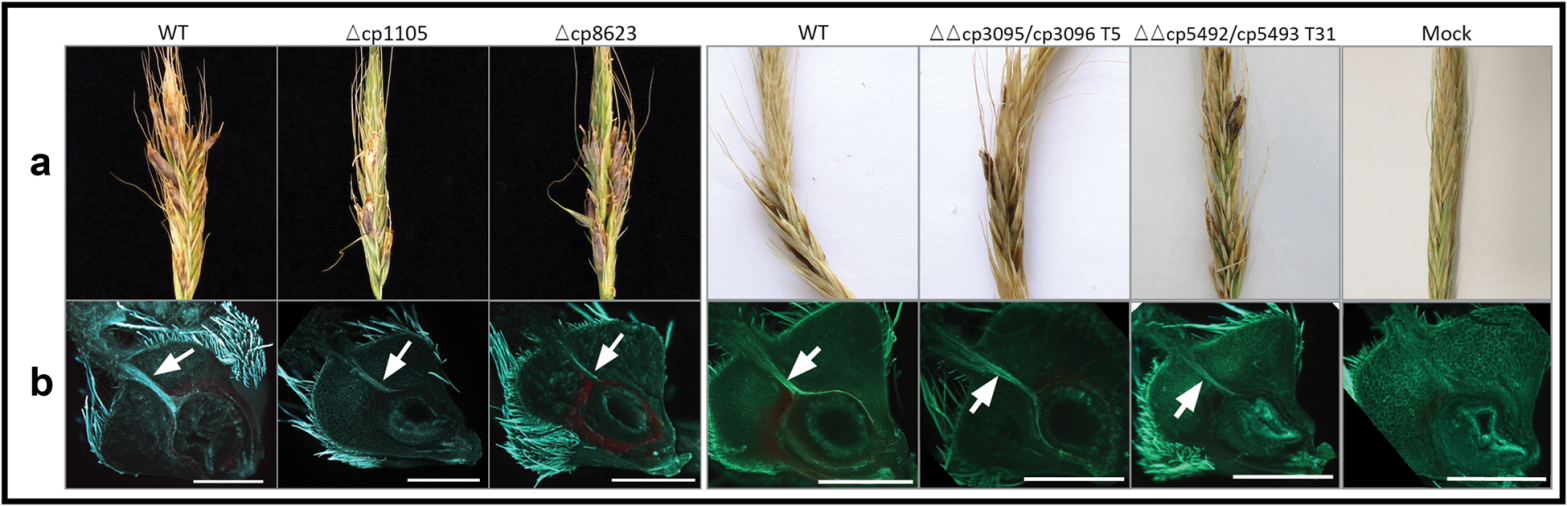
Pathogenicity assays using the *C. purpurea* wild-type *Cp*20.1 strain and the Δcp1105 and Δcp8623, ΔΔcp3095/cp3096 and ΔΔcp5432/93 deletion mutants. **a** Rye ears were infected with conidia suspensions of the different strains, each deletion strain was simultaneously infected and compared to the wild-type. Honeydew formation and sclerotia formation were monitored for 3 weeks. **b** *In vitro* cultivated rye florets infected with the different strains. After 6 days, cross-sections of the ovaries were stained with aniline-*blue* (which emits *green* fluorescence), allowing the detection of fungal hyphae (indicated by *arrows*) within the plant tissue by fluorescence microscopy. The infection routes of the deletion strains are similar to those of the wild-type (bars: 1 mm)

To test the impact of the deletion of the effector candidates on the virulence of *Cp*20.1, pathogenicity assays were performed on intact rye plants and the infection pathway was analyzed using dissected rye ovaries (Fig. 8). Of 22 rye plants inoculated with Δcp1105, 15 showed honeydew production as well as sclerotia formation. Thus, the infection rate of Δcp1105 is reduced to 68% compared to the wild-type (100%). The amount of honeydew per infected ovary as well as the sclerotia appeared to be wild-type like (Fig. 8; an exact quantification is not possible). However, the induction of honeydew formation was delayed (9–20 dpi) compared to wild-type infections (7–8 dpi).

In total, 23 rye ears were inoculated with Δcp8623, leading to infection signs in 16 ears, making up an infection rate of 70%. The formation of honeydew was initiated around 7–9 dpi, thus appeared to be wild-type like.

Five rye ears were inoculated with ΔΔcp3095/cp3096, honeydew formation became visible 7–8 dpi and thus the infection rate is wild-type like. Also, sclerotia formation of ΔΔcp3095/cp3096 is comparable to *Cp*20.1 (Fig. 8). 22 rye ears were inoculated with the double deletion strains of *cp5492/cp5493*, the occurrence of disease symptoms was also comparable to simultaneously wild-type inoculated ears.

Microscopical analyses of dissected ovaries inoculated with Δcp1105, ΔΔcp3095/cp3096, ΔΔcp5492/cp5493 and Δcp8623 were indistinguishable from wild-type infections (Fig. 8).

Taken together, the deletion strains of *cp1105* and *cp8623* have reduced virulence. Both have a reduced infection rate and Δ1105 also has a retarded infection process. The infection process of the double deletion strains ΔΔcp3095/cp3096 and ΔΔcp5492/cp5493 are wild-type like. Sclerotia formation and the infection route are wild-type like for all deletion mutants.

## Discussion

The ergot disease is tightly interwoven with history of mankind and agriculture since the Middle Ages. Especially in the past decades knowledge about the disease was increased but the underlying mechanisms are still poorly understood. One hallmark of the infection process is the avoidance of drastic host defense reactions during tissue colonization, instead plant cells in direct contact of *C. purpurea* hyphae remain healthy [36, 37] and in wild-type infections no oxidative burst is detectable [19, 20]. One long-standing theory is that *C. purpurea* mimics pollen tube growth within rye ovaries to avoid massive host-defense reactions. However, knowledge about the molecular mechanisms underlying this highly complex infection pattern was limited. The dual RNA-Seq approach applied here for the first time allows the analysis of the transcriptomes of both the pathogen and the host simultaneously. Besides wild-type infected rye ovaries and mock-treated controls, ovaries infected with the two mutants Δcptf1 and Δcpcdc42, strains which are virulence-attenuated at different infection stages, were included.

On the pathogen-side, only the transcriptomes of *Cp*20.1 and Δcptf1 could be evaluated as the proportion of fungal biomass and thus the proportion of fungal reads in the Δcpcdc42 sample were not sufficient. In future experiments which are based on *C. purpurea* strains with reduced colonization phenotypes, enrichment strategies should be performed, using only defined parts of the ovaries e.g. the stigmatic hairs or laser dissection of infected tissue. For the plant transcriptomes as well as for the *Cp*20.1 and Δcptf1 transcriptomes the quantity of reads was sufficient and our very conservative data evaluation resulted in the identification of 112 (plant) and 48 (fungus) differentially expressed genes. The output is comparable to other dual RNA-Seq approaches of fungus-plant interactions at early infection stages as for example the transcriptomic analysis of sugarcane infected with *Sporisorium scitamineum* (5 dpi, 3 biological replicates) resulted in the identification of 125 differentially expressed genes [38]. The differentially expressed genes identified in our study included already known virulence factors. Furthermore, expression analysis of selected candidates from independent biological replicates confirmed the obtained results, together underlining the robustness of our data.

Principally, our data show that the infection with *Cp*20.1 causes alterations in the host transcriptome, including both, up- and downregulation of pathogenesis-related genes. Thus, rye plants obviously recognize the infection, but no effective defense reactions seem to be established. Hence, the symptomless *in planta* growth of the fungus is not due to non-recognition because of “pollen-tube mimicry” but must have other reasons. One putative explanation might be a partially mutualistic mode of the *C. purpurea*-rye interaction which has been discussed earlier [39, 40]. *C. purpurea* causes only limited damage to the host, as only single ovaries of rye plants are infected and unlike Fusarium head blight, ergot infection does not spread from spikelet to spikelet [41, 42]. Furthermore, the toxic ergot alkaloids produced by the fungus *in planta* convey a feeding protection against grazing animals. Additionally, this hypothesis is supported by the close taxonomic relationship of *C. purpurea* to major endophytes like *Epichloe* species. Taken together, the absence of drastic defense reactions by the host might depict a trade-off to benefit from *C. purpurea* infections.

Interestingly, the virulence attenuated mutants induce the expression of other partially overlapping defense-related genes. In particular, the upregulation of a chitinase gene (Sc6Loc01460943.1) is indicative for an ongoing basal defense response against these mutants upon PAMP detection. If this reaction is due to general defects of the mutants or if they lack proteins like Avr4 in *Cladosporium fulvum* that shield the fungal cell wall from chitinases [43] remains to be elucidated.

Another interesting candidate in the host transcriptomes is the xylanase-inhibitor (Sc4Loc00580338.2) which is upregulated in Δcptf1 infected-ovaries. The homologous protein of wheat inhibits fungal endo-1,4-beta-D-xylanases [44] and thus is most likely involved in plant defense. Earlier studies already revealed that *C. purpurea* secretes xylanases during the entire infection process [30]. Accordingly, *xyl2* (*cp8536*) is highly expressed during the infection process of *Cp*20.1 and *xyl1* (*cp3570*) is significantly down regulated in Δcptf1 (Additional file 6, No.23; Fig. 5; [30]). Therefore, it was even more surprising that neither deletion of *xyl1* nor double deletion of both xylanases (*xyl1*/*xyl2*) significantly affected virulence of the fungus [39, 30]. The presence of a xylanase-inhibitor which inactivates the fungal proteins might however explain this phenomenon.

Besides differences between the plant transcriptomes, all three data sets of “infected” ovaries have a significantly induced auxin response in common. For example, in *Cp*20.1 infected ovaries expression of Sc1Loc01149658.2 is induced. The corresponding protein has homology to proteins of the auxin-responsive AUX/IAA family, which as the name already suggests, has been identified due their auxin-dependent expression and many of these family members are involved in the regulation of auxin-induced expression in plants [45]. Similarly, an induced expression of four genes encoding proteins with homology to 5NG4 has been detected in Δcpcdc42 infected ovaries. The protein was first identified in loblolly pine and is conserved in evolutionary distant species. It is supposed to be a transmembrane protein with transporter functions and its expression is highly auxin-inducible [46]. Furthermore, in *Cp*20.1 and Δcptf1 infected ovaries two genes probably encoding indole-3-acetic acid-amido synthetases (Sc3Loc01478542.1 and Sc2Loc00096015.6) are significantly upregulated. These proteins are known to conjugate amino acids to the main auxin indole-3-acetic acid (IAA) [47]. IAA-conjugates are less active and depending on the amino acid, they are considered to be either storage or catabolism conjugates. However, more recent data show, that at least the IAA-Asp plays an additional role in pathogenesis. In *A. thaliana* plants infected with *Botrytis cinerea* or *Pseudomonas syringae* expression of the auxin-conjugating GH3.2 is induced leading to the accumulation of IAA-Asp which promotes disease development and induces expression of different virulence genes in the pathogens. This disease promoting effect is not restricted to *A. thaliana* and might depict a common mechanism [48] which could also apply for the *C. purpurea*-rye interaction.

Moreover, all mentioned auxin-related genes are usually required or known to be upregulated under auxin access. However, the expression of rye auxin biosynthesis genes is not induced under the tested conditions. On the contrary, recent findings prove that *C. purpurea* secretes significant amounts of auxins (P. Galuszka, pers. communication) as well as the closely associated cytokinins [17, 18]. Hence, the fungus might hijack the auxin/cytokinin homeostasis by direct secretion of these plant hormones *in planta* to facilitate infection. Though auxins and cytokinins are mainly known as developmental hormones they also affect a multitude of defense reactions. In general, auxin signaling acts in an opposing manner to SA and for example represses PR gene expression, while it has overlapping functions with JA-induced defense (reviewed by [49]). Furthermore, auxin/cytokinin signaling might depict an attractive target especially for invading biotrophic pathogens due to its involvement in nutritional signaling and senescence [50–52].

Another potent strategy to alter the host in a pathogen advantageous way is the secretion of effector proteins. For this reason, we performed a bioinformatic analysis of the recently available *C. purpurea* genome revealing that the fungus comprises the potential to encode for 470 effector candidates (with a size of 10–330 aa). A less stringent approach performed by [6] resulted in a set of 726 candidate genes in *C. purpurea* which is comparable to other facultative biotrophic pathogens. Their comparison of secretomes of fungi with different lifestyles furthermore revealed that biotrophic fungi including *C. purpurea* contain more effector candidates without functional annotations than necrotrophic fungi [6]. Our transcriptomic analyses now show that many of the identified effector candidate genes are indeed highly expressed during the infection process making up 17% of the 150 most highly expressed genes. Five candidates even belonged to the top10 most highly expressed genes of *Cp*20.1. Moreover, seven candidates are downregulated in the virulence-attenuated mutant Δcptf1. Generally, the identification of fungal effectors has been focused on biotrophy-specific expression [53, 54] and for example a similar enrichment of secreted proteins was observed in the transcriptome of *Magnaporthe oryzae* comparing invasive hyphae and mycelium from axenic culture [55]. Based on our expression data, the six candidates, *cp1105*, *cp5492*, *cp5493*, *cp3095*, *cp3096* and *cp8623*, were chosen for detailed analyses to gain deeper insight into the *C. purpurea* effector secretion.

Firstly, the expression data of the RNA-Seq analysis could be confirmed by qRT-PCR. All six genes were highly expressed 5 dpi compared to the housekeeping genes. Furthermore *cp1105*, *cp5492*, *cp5493, cp3095* and *cp3096* showed the typical expression pattern of effectors, as their expression is induced in the early infection stages (3 or 5 dpi), compared to the expression in the later infection stage (10 dpi) and in liquid medium. Interestingly, *cp1105*, *cp5493, cp3095* and *cp3096* are most highly expressed during the very early infection stage analyzed here (3 dpi), while expression of *cp5492* peaks slightly delayed at 5 dpi. This indicates the involvement of different regulatory mechanisms for the effector candidates even within the small effector cluster. Furthermore, it might indicate different functions during infection: in the initial phase the host-pathogen interaction is established, while around 5 dpi *C. purpurea* growth switches from restricted polar growth to colonization of plant material. Thus, *cp5492* might be important for this developmental step *in planta*. Differential regulation and functions of clustered effectors were also observed in the biotrophic pathogen *U. maydis* [56]. In the later infection stages, 10 dpi, the expression rates of these effector candidates clearly decreased. This is in line with the typical infection pattern of *C. purpurea*, as the plant material is almost entirely replaced by the fungal sphacelium and the production of sclerotia is initiated at this infection stage. Thus, the fungal development *in planta* is nearly completed and the secretion of effectors to dampen host defense responses seems no longer to be necessary.

The expression pattern of *cp8623*, in contrast, differs considerably and does not follow the effector expression pattern. The irregular expression *in planta* as well as the transcriptional induction in axenic culture, points towards an (so far unknown) environmental factor influencing the expression of this gene. *C. purpurea* faces constantly changing conditions during the infection process with regard to pH and nutrient availability [57–59]. If one of these factors directly influences the expression of *cp8623* needs to be further investigated. The identification of the protein function could certainly contribute to the clarification of this regulatory mechanism. An indication for Cp8623 function is given by the predicted LysM domain of the protein. LysM domains are carbohydrate-binding domains which are widespread within the fungal kingdom [60]. Comparison of 403 fungal lysM-containing proteins resulted in the classification of these proteins according to their overall domain architecture. Like Cp8623, most of the proteins do not contain any additional motif besides a signal peptide and harbor only one lysM domain. The best known examples for these so called “LysM effectors” are the virulence factors Ecp6 from *C. fulvum* (containing 3 LysMs) and Slp1 from *M. oryzae* (containing 2 LysMs) which sequester chitin oligosaccharides intervening in chitin-triggered PTI [43, 60–62]. Thus, the decreased virulence of Δcp8623 might be due to chitin-triggered defense responses. However, like in *C. fulvum* further mechanisms to shield *C. purpurea* hyphae from plant chitinases must exist as Δcp8623 is still able to colonize the plant. If Cp8623 is indeed involved in chitin-sequestration a localization of the protein at the host-pathogen interface would be expected as described for Slp1 [62].

Beside the expression it would have been informative to identify the localization of the effector candidates, as this might have given hints towards their possible function. In general, a preferential localization of effectors within the host apoplast is expected for *C. purpurea* as it mainly grows intercellularly. We applied a mCherry fusion approach for all effector candidates to observe their localization *in planta.* This included the addition of nuclear sorting signals which would allow the identification of translocation of candidates into the plant cells [32]. Specific fluorescence could only be observed for Cp1105::mCherry in axenic culture. The accumulation of the protein in the media surrounding the hyphae in axenic cultures is in contrast to the qRT-PCR data which indicate low expression rates for *cp1105* under these conditions as the fusion constructs were under control of the native promoter. The expression levels were retested under axenic growth conditions and the expression level of *cp1105* was several fold increased in the construct harboring strains. Obviously, the ectopic integration of *cp1105* deregulated the overall expression of *cp1105* significantly and despite the use of the native promoter, the strength of the fluorescence does not allow conclusions about the native expression. Despite the altered expression level, observation of the fluorescence of the surrounding medium points to an apoplastic localization of Cp1105, congruent with the expected preferential localization of effectors in the host apoplast. Considering the altered hyphal morphology of the Δcp1105 mutant emphasizes a role of the corresponding protein in cell wall organization. Most likely, the reduced growth rate and thus the delayed infection of rye ovaries is a direct consequence of the undulated morphology of the hypha. Remarkably, Cp1105 contains 7 Cys-residues within the processed protein and as disulphide bonds have been shown to be of special importance for the stability and function of effector proteins this might indicate a complex folding mechanism for this protein [63]. The precise function of Cp1105 remains open to speculation. The altered hyphal morphology could suggest a stabilizing function of the protein like described for the putative matrix protein Bas4 of *M. oryzae* [55] or RTP1 of rust fungi [64, 65].

Testing of the other fusion constructs did not allow reproducible observation of specific fluorescence, neither *in planta* nor in axenic culture. The most likely reason is that the concentration of the protein is not sufficient to emit detectable fluorescence, especially *in planta*. Despite the expected induced expression the protein is diluted in the host apoplast and the surrounding host cells have considerable background fluorescence that likely covers specific fluorescence. This hinders detection of specific fluorescence and distinguishing of samples with fusion construct harbouring strains from wild-type samples. Similar problems occurred in other systems, e.g. in *U. maydis*/maize where fluorescence based observation of effectors failed [66]. For *U. maydis*, an alternative approach was established which is based on intracellular biotinylation of translocated effectors and thus, enabling conclusions about their localization and targets [67]. However, this approach requires genetic modification of the host plant which is not available for rye. For future experiments, alternative strategies for determination of the *in planta* localization have to be evaluated.

A compatible interaction of *C. purpurea* and rye is assumed to be tightly regulated which involves fungal effectors. To finally determine the individual contribution of the effector candidates to this interaction, deletion strains of the respective genes were studied especially in regards of affected virulence.

To avoid redundant effects in knock-out strains, we performed double deletions when neighboring genes of candidates also matched the previously mentioned effector features. This applied to *cp5493* and *cp3095. cp5493* forms a small effector cluster with *cp5492* and *cp3095* is the first gene of a larger cluster with five effector candidates interrupted by one non-effector candidate gene. The two genes in the small cluster do most likely not originate from recent duplication as they share no significant similarity on nucleotide level and though both are highly induced *in planta*, they are not under the control of the same promoter. We generated three independent deletion strains, and all of them were not retarded in the infection process. The findings about the deletion strains in the course of this study tempt us to assume that at least the gene products of *cp1105* and *cp8623* are involved in the infection process although the individuals are dispensable. Though, their function either just slightly accelerates the infection process or it takes the fungus a few days to sense the irregularity of the typical infection process but it is able to countervail, e.g. by induction of other effectors.

We refrained from deletion of the complete cluster and limited the approach to *cp3095* and *cp3096* as *cp3097* is the non-effector candidate and deletion of more than two genes has not been successfully performed in *C. purpurea* before. However, partial deletion of this effector cluster did not affect virulence. As the remaining cluster genes show high similarity to the deleted ones (54–90%) they could probably compensate for any putative loss of function. The individual deletion of effectors does not severely affect the virulence of *C. purpurea*, as it has been observed for other biotrophic fungi [63, 68, 69]. It is becoming more and more evident that the network of effectors is a highly complex and often redundant system (reviewed in [7, 54]). Instead, more severe phenotypes with regard to virulence are observed when complete effector clusters or transcription factors controlling several effectors or SSPs are deleted, such as *sge*1 homologues in a.o. *Fusarium oxysporum*, *Zymoseptoria tritici* and *U. maydis* [56, 70–72]. Sge1 homologues in *C. purpurea* are currently investigated but our previous and present findings indicate that Cptf1 regulates effectors as well. The previous focus while studying *cptf1* was on ROS regulation and it was shown that during the infection with Δcptf1 the host cells induced an oxidative burst [19]. This effect was ascribed to reduced fungal ROS quenching but neither deletion of a fungal superoxide dismutase nor of a secreted catalase did affect virulence [29, 73]. The plant oxidative burst could also be activated upon recognition of the fungal infection due to the downregulation of effectors in Δcptf1. This assumption is supported by this study which indicates significant downregulation of several effector genes in Δcptf1 *in planta*. Consequently, we assume that Cptf1 regulates effectors in *C. purpurea* and that this contributes to the virulence attenuation of Δcptf1. For effector research this means that severe virulence attenuation only occurs when several effectors are affected but not upon individual deletion. Based on these findings we can speculate that the absolute essentiality of establishing a compatible interaction with the host plant drove the development of very robust infection mechanisms in *C. purpurea*. The robustness is very likely based on effectors with overlapping functions and compensation mechanisms. This system itself hinders unveiling of specific functions or targets of individual proteins as it is unlikely to have those simple relations. As a biotrophic pathogen *C. purpurea* has likely evolved several strategies to avoid host cell death. How successful *C. purpurea* performs at keeping host cells alive can be observed on the ovarian cap. It is separated from the ovarian base in the process of infection but despite the separation from the feeding base the tissue is mainly intact and stays alive for a while [37]. Even the development of viable grains on top of sclerotia has been described [74]. At this stage of investigation it is unclear how the fungus manipulates the tissue but this is a function typically ascribed to effectors [5–7]. An alternative mechanism of plant pathogens is the manipulation of phytohormone signaling. As recently published from our group, *C. purpurea* synthesizes the phytohormones cytokinins which are known to inhibit senescence in plants [17, 18, 75]. “Classic effectors” and phytohormone signaling could provide two very different mechanisms in *C. purpurea* both aiming at keeping the host alive. Furthermore, we have to take into account the broad host range of *C. purpurea*. In contrast to other well-studied biotrophic fungi such as *U. maydis, M. oryzae and Blumeria graminis, C. purpurea* is not restricted to one host species or genus but is able to infect more than 400 grass species [76]. This of course requires more flexibility with regard to infection strategies. One infection mechanism and the mediating effectors might be of higher importance in specific host-*C. purpurea* interactions. Similar to organ specific effectors in host-restricted fungi [77, 78], *C. purpurea* as an organ-restricted pathogen might have host specific effectors which functions only become obvious in few interactions. To clarify this, the suitability of alternative model host plants should be explored and included in future experimental designs. Beside the broad host range, the system is further complicated by the fact that *C. purpurea* infection might also provide benefit for the host plant [40]. In the course of evolution this could lead to increased tolerance towards *C. purpurea* infection even despite the lack of individual effectors. As possible host plants are diverse and only distantly related this does not have to apply to all host-*C. purpurea* interactions. This further emphasizes the importance of the inclusion of alternative host plants.

This study represents the first transcriptome sequencing approach on a host-diverse biotrophic fungus combined with functional characterization. The results show that *C. purpurea* is visible to the host rye and that the host plant’s phytohormone signaling is altered. The obtained data set for fungal expression shows that several effectors are regulated by Cptf1 and points to effectors that participate in the infection process although functional characterization showed that the individual is dispensable. Considering the lifestyle of *C. purpurea* these findings underline the biotrophic nature and the robustness of the *C. purpurea* infection mechanism and the difficulty of unriddling the complex effector network. For a deeper understanding the effector targets should be addressed. Yeast-two-hybrid screens using cDNA libraries of host plants, or interorganismic pull downs are promising methods to determine the targets of the proteins within the plant tissue. For determination of *in planta* localization the potential of other approaches should be assessed as tagging with fluorescent proteins is not applicable for *C. purpurea*. Secondly, the effector cell-to-cell movement described for other fungi should be investigated in particular with respect to the host-pathogen interface build up at the ovarian axis. Hyphae stop spreading into the plant tissue at this point and the question remains if this is also true for effector proteins. Additionally, the use of an alternative host species would help to gain insights into *C. purpurea*-host interaction, especially if a genetically modifiable plant such as *Brachypodium distachyon* is used [79]. This will in turn promote effector research in *C. purpurea* a cut above, which is the identification of the precise function in the interaction with the host.

## Conclusions

*C. purpurea* infects a broad range of grass species in an organ-restricted manner without the induction of obvious defense symptoms. In contrast to well-studied biotrophic fungi, *C. purpurea* infection is organ specific but not host species restricted. Our results point to an intensive cross-talk between the host and the fungus and this study gave a first insight into this system, revealing its complexity. The unique lifestyle of *C. purpurea*, especially the broad host range and the putative beneficial effect of infection on host plants, likely evolved a robust infection mechanism that is integrated in sophisticated host-pathogen interaction. We assume that *C. purpurea* has several mechanisms with overlapping aims to enable the biotrophic infection such as phytohormone signaling and effector secretion to keep host cells alive. The findings of this study provide a good basis to design future approaches to unravel those mechanisms and gain a deeper and more detailed understanding.

## Methods

### Sequencing and processing of RNA-Seq data

All four RNA samples were processed by BGI-Hongkong Co. (Hong Kong). After size selection (200 – 700 nucleotides) and PCR amplification the fragments were sequenced with a paired-end protocol on an Illumina HiSeq™ 2000 sequencer. After quality control (Q ≥ 20) the cleaned reads were mapped to “de novo” assembled transcripts. The transcripts were sorted into putatively plant, putatively fungal transcript and transcripts of unclear origin via blast searches against the *C. purpurea* genome (http://pedant.gsf.de/pedant3htmlview/pedant3view?Method=analysis&Db=p3_p76493_Cla_purpu), NCBI databases and a collection of rye cDNA contigs [25]. BGI Hongkong Co. removed all reads mapping as well to “fungal” as to “plant” de novo assembled transcripts from the clean reads. The cleaned and sorted reads - as provided by BGI - are deposited in the NCBI SRA archive (accession number SRP091705). Advance access to the *S. cereale* Lo7 genome sequence enabled us to confirm 94% of the “plant” de novo assembled transcripts and to analyse the differential gene expression on the basis of both genomes – *C. purpurea* and *S. cereale*. Principal component analysis of the four samples was done by R script [80] using the FPKM values of the reads matching the “plant” and “fungal” de novo assembled transcripts. For gene ontology (GO) analysis three programs were used: 1) Blast2Go Basic [81], 2) CateGOrizer with classification method set to “GO_slim2” and with occurrence counting set to “consolidated single occurrences” [82] and 3) REVIGO [83]. The quality of all left reads was checked via FastQC 0.10.1 (http://www.bioinformatics.babraham.ac.uk/projects/fastqc/). TrimGalore 0.3.3 (parameters: −-quality 30 --phred64 --stringency 13 -e 0 --length 30 --paired; http://www.bioinformatics.babraham.ac.uk/projects/trim_galore/) was used to gather all reads with a minimal length of 30 bases and a Q-value ≥ 30. Blast2Go Basic was used to update the annotation of ergot genes and to annotate rye genes. Rye genes were further annotated via blasts against “Reference Enzymes 3.1” and “PlantCyc enzymes” in the Plant Metabolic Network (http://www.plantcyc.org), which gives also detailed pathway informations.

Differential gene expression analysis basically followed alternate protocol B “Quantification of reference annotation only” [84]. The reunited fungal and plant reads of each RNA sample were mapped to the genomes via TopHat (parameters: −-no-discordant --no-mixed –GTF). Multi-mapped reads and PCR duplicates were removed via SAMtools (samtools view –b –q 5 and samtools rmdup) [85]. Pairwise comparisons were made with Cuffdiff (part of Cufflinks) for rye (mock - *Cp*20.1, mock - Δcptf1 and mock – Δcpcdc42) and ergot (Δcptf1 - *Cp*20.1; mitochondrial genes masked) [84, 86–88]. As there are no replicates automatically the dispersion method “blind” was applied, meaning that “all samples were treated as replicates of a single global condition”.

### Screening for effector candidates in *C. purpurea* genome

All *C. purpurea* predicted proteins with a size of 10–330 amino acids were screened with the programs SignalP4.0, SigCleave, Phobius 1.01 and TargetP 1.1 for signal peptides [89–92]. If TargetP predicted a mitochondrial localization and either WolF PSORT or Yloc confirmed this prediction [93–95], the protein was excluded from the pool of candidate effectors. All proteins with a signal protein according to three out of the four signal prediction programs were then screened for transmembrane domains (TMs). All candidates which might possess no TM according to two out of three programs used (Phobius, TMHMM 1.0 and DAS) were kept in the pool if they were without GPI-anchor (fragAnchor) or without an ER signal (ScanProsite, searched motif: PS00014) [96–100]. They were accepted into the final pool of effector candidates – without regard of the number of cysteines in their sequence and their potential for disulfide binds, as not all biotrophic fungal effectors found and investigated so far fulfill all criteria described for effectors. Proteins which did not match the “3 out of 4 programs predict a signal peptide” rule joined the final pool, if they contained at least 2 cysteines and at least one disulfide bond was predicted by either DiANNA 1.1 or DISULFIND 1.1 [101–104]. Also proteins, which contained TMs according to only one predicting program, were taken in the final pool, if they contained at least two cysteines and one predicted disulfide bond.

### Strains, media and growth conditions

The wild-type *Claviceps purpurea* (Fr.) Tul. strain 20.1 (*Cp*20.1,[105]), a putatively haploid (benomyl-treated) derivative of the standard field isolate T5 (Fr.:Fr.) Tul. isolated from *Secale cereale* L. (Hohenheim, Germany), was used for the generation of mutants and as the wild-type control in all experiments. Mycelia were grown on complete medium BII [106] for cultivation and DNA isolation. Conidia were obtained from mycelia cultivated on Mantle medium [107]. All strains were cultivated in the dark at 26 °C. Vector construction using the yeast recombinational method was performed in the yeast strain FY834 [108].

### DNA extraction and analysis

Standard recombinational DNA methods were used as described previously [109, 110]. Genomic DNA from *C. purpurea* was prepared from lyophilized mycelia according to Cenis [111]. PCR was performed as described in [110] using BioTherm Polymerase (GeneCraft). PCR amplifications of fusion proteins or complementation fragments were performed using the proof reading Phusion polymerase (Finnzymes). All primers used are listed in Additional file 13 and were synthesized by Biolegio (Nijmegen). Southern blotting was performed using Hybond-N^+^ nylon filters (Amersham) according to the manufacturer’s protocol. Filters were hybridized using [α-^32P^]-dCTP-labelled probes. DNA sequencing was carried out as described in [29]. Protein and DNA sequence alignment, editing and organization were done with DNA Star (Madison). Further sequence analyses were performed using BLAST at the National Center for Biotechnology Information, Bethesda, MD, USA [112].

### Replacement and complementation vector construction

All vectors were generated using the yeast recombinational cloning method [113]. Complementation and fusion protein vectors were constructed based on the described vector system [34, 114]. Cp5492/cp5493: The flanking regions of *cp5492/cp5493* were amplified with the following primers containing overlapping sequences towards the yeast shuttle-vector pRS426 or the hygromycin resistance cassette: 5F_5492 (*Xho*I)/5R_5492 for the 5′-spanning region (1083 bp); 3F_5493/3R_5493 (*Xho*I) for the 3′-spanning region (917 bp). The PCR-products, the linearized yeast shuttle vector pRS426 [113] and the hygromycin resistance cassette (amplified with primers hph_F/hph_R 1433 bp) were transformed into yeast strain FY834 for homologous recombination. DNA was isolated from yeast cells using the SpeedPrep yeast plasmid isolation kit (DualSystems) and transformed into *E. coli*. After DNA isolation, restriction with *EcoR*I and *Xho*I resulted in an 1871 bp fragment (5′ split fragment) and the 3’split fragment (2059 bp) was obtained by restriction with *Xho*I and *Sac*II. Equal amounts of the fragments were used to transform the 20.1 strain of *C. purpurea* (Additional file 11). Cp3095/cp3096: Replacement vector construction was performed in the same way as for cp5492/cp5493, using the primers 5F_3095 (*Xho*I)/5R_3095 for the 5′-spanning region (913 bp); 3F_3096/3R_3096 (*Xho*I) for the 3′-spanning region (880 bp). Restriction with *EcoR*I and *Hind*III resulted in a 1720 bp fragment (5’ split fragment) and the 3’split fragment (2027 bp) was obtained by restriction with *Hind*III and *Sac*II. Equal amounts of the fragments were used to transform the 20.1 strain of *C. purpurea* (Additional file 11). Cp1105: Replacement vector construction was performed in the same way as for the deletion of *cp5492/cp5493* except that a phleomycin cassette was used (Cpble_F1/Cpble_R1, 1853 bp) instead of the hygromycin cassette. The flanking regions were amplified with the primers 5F_1105 (*EcoR*I)/5R_1105 for the 5’flanking region (1000 bp) and 3F_1105/3R_1105 (*EcoR*I) for the 3’flanking region (906 bp) of *cp1105*. The 3789 bp replacement fragment was excised by restriction with *EcoR*I and used to transform the 20.1 strain of *C. purpurea* (Additional file 10). Cp8623: Primers 5F_8623 (*EcoR*I)/5R_8623 and 3F_8623/3R_8623(*EcoR*I) resulted in 875 bp (5’flanking region) and 963 bp (3’flanking region). The 3719 bp replacement fragment was excised by restriction with *EcoR*I and used to transform the 20.1 strain of *C. purpurea* (Additional file 10).

### Fungal transformation

Protoplasts were prepared from *C. purpurea* strains as described previously [115]. Integration events were confirmed by diagnostic PCR using specific primers as indicated (Additional file 13). Single spore isolation was carried out to obtain homokaryons of putative transformants. Additionally, Southern blot analyses were performed with deletion mutant to confirm single integration events.

### RNA extraction and quantitative RT-PCR

For detection of fungal and plant gene expression, cDNA of infected and uninfected *in planta* material was analyzed. Ovaries for RNA isolation were shock-frozen with liquid nitrogen, lyophilized and ground with a mortar. Total RNA was isolated using the RNeasy Midi Kit (Qiagen). Prior to sequencing, RNA quality was analysed using Agilent 2100 Bioanalyzer (Agilent Technologies). Reverse transcription-PCR was performed using the Superscript II (Invitrogen) and 2 μg of total RNA as a template, according to the manufacturer’s instructions. cDNA was tested for DNA contamination using primers (pls1_fw/rev) spanning an intron. qPCR reactions were performed with the BioRad iQ SYBR Green Supermix and the iCycler Thermal Cycler (BioRad). Programming, data collection and analyses were performed with the iCycler iQ Real-Time Detection System Software Version 3.0 (BioRad). The expression of all tested fungal genes was normalized to the expression of genes encoding β-tubulin (CCE34429.1; Tub_uni/Tub_rev), γ-actin (AEI72275.1; Actin_uni/Actin_rev) and glyceraldehyde-3-phosphate dehydrogenase (X73282.1; Gpd_uni/Gpd_rev) as described previously [116]. The expression of all tested plant genes was normalized to the expression of genes encoding the ADP-ribosylation factor (wheat unigene Ta2291; ADP_fw/ADP_rev), the cell division control protein, AAA-superfamily of ATPases (wheat unigene Ta54227; CDC_fw/CDC_rev) and RNase L inhibitor-like protein (wheat unigene Ta2776; RLI_fw/RLI_rev) modified after [117].

### Plant growth conditions, infection, and sampling

Pathogenicity assays were performed using the cytoplasmic male sterile *Secale cereale* Lo37‐PxLo55‐N (KWS Lochow GmbH) cultivar, which was cultivated in growth chambers under conditions of 15 h light (8,000 Lux; 16–18 °C)/9 h darkness (13–15 °C). Before planting, seeds were stratified for 5–6 weeks at 1–2 °C and vernalization took place in soil/compost 3:2 at 14–15 °C (day) and 9–10 °C (night) with 9 h light (6,000‐10,000 Lux). For *in planta* pathogenicity assays, florets of blooming ears (30-40/ear) were inoculated with 5 μL of a suspension containing about 10^6^ ml^−1^ conidia collected from Mantle agar, as described in [118]. To avoid cross contamination, the ears were covered with paper bags directly after inoculation. The *in vitro* pathogenicity assay was performed as described in [119].

### Microscopic analyses

For microscopic studies *in planta*, rye ovaries were stained with KOH-aniline-blue as described previously [119] and examined with a Zeiss DiscoveryV20 stereo microscope fitted with an AxioCam MRc camera. Fluorescence microscopy was performed with a Zeiss AxioImager M1 microscope. mCherry fluorescence was examined using the filter set 43 HE Cy 3 shift free (excitation BP 550/25, beam splitter FT 570, and emission BP 605/70). Images were captured with a Zeiss AxioCam MRm camera Image analysis was performed with Axiovision Rel 4.8 software.

### Gene accession

All fungal genes from this study are listed with a shorthand, complete gene identifiers and NCBI accession numbers in Additional file 14 . For the rye genes, gene identifiers have been used according to the webblast (http://webblast.ipk-gatersleben.de/ryeselect/).

## Additional files

Additional file 1: Technical Data of RNA-Seq (XLSX 11 kb)

Additional file 2: Plant genes differentially expressed after infection with *Cp*20.1, Δcptf1, Δcpcdc42, mock. (XLSX 39 kb)

Additional file 3: GO term distribution of *in planta* expressed *Cp*20.1 genes (XLSX 8 kb)

Additional file 4: Overview of GO terms “biological process” for *in planta* expressed *Cp*20.1 genes (XLSX 17 kb)

Additional file 5: REVIGO scatterplots of biological process GO terms associated with *Cp*20.1 genes of high (A), medium (B) and low (C) *in planta* expression. (PDF 165 kb)

Additional file 6: 150 most highly expressed fungal genes (XLSX 32 kb)

Additional file 7: Fungal genes controlled by Cptf1 (XLSX 18 kb)

Additional file 8: Predicted *Claviceps purpurea* effectors (XLSX 106 kb)

Additional file 9: Microscopical analyses of strain *Cp*20.1 mCherry:NLS (PDF 491 kb)

Additional file 10: Deletion strategy of *cp1105* and *cp8623* and identification of deletion strains (PDF 281 kb)

Additional file 11: Deletion strategy of *cp3095/cp3096* and *cp5492/cp5493* and identification of deletion strains (PDF 209 kb)

Additional file 12: Growth assay of Δ1105 and Δ8623 (PDF 43 kb)

Additional file 13: Oligonucleotides used in this study (PDF 82 kb)

Additional file 14: NCBI Accession numbers of fungal genes (XLSX 19 kb)

## Abbreviations

Aa: Amino acids
bp: Base pairs
CAZymes: Carbohydrate active enzymes
Cys: Cystein
dpi: Days post inoculation
ETI: Effector triggered immunity
ETS: Effector triggered susceptibility
HR: Hypersensitive response
kb: Kilo base pairs
PAMP: Pathogen associated molecular patterns
PCA: Principal component analysis
PR: Pathogenesis related
PRR: Pattern recognition receptors
PTI: PAMP triggered immunity
qRT-PCR: Quantitative reverse transcriptase-PCR
R: Plant disease resistance
ROS: Reactive oxygen species
SP: Signal peptide
