# Supplementary material for: Cross-talk of the biotrophic pathogen Claviceps purpurea and its host Secale cereale
Source: BMC Genomics. 2017 Apr 4;18:273. doi: 10.1186/s12864-017-3619-4 (PMC5379732; doi:10.1186/s12864-017-3619-4)
Supplement: Supplementary file 5 — REVIGO scatterplots of biological process GO terms associated with Cp20.1 genes of high (A), medium (B) and low (C) in planta expression. (PDF 165 kb) [file 12864_2017_3619_MOESM5_ESM.pdf]

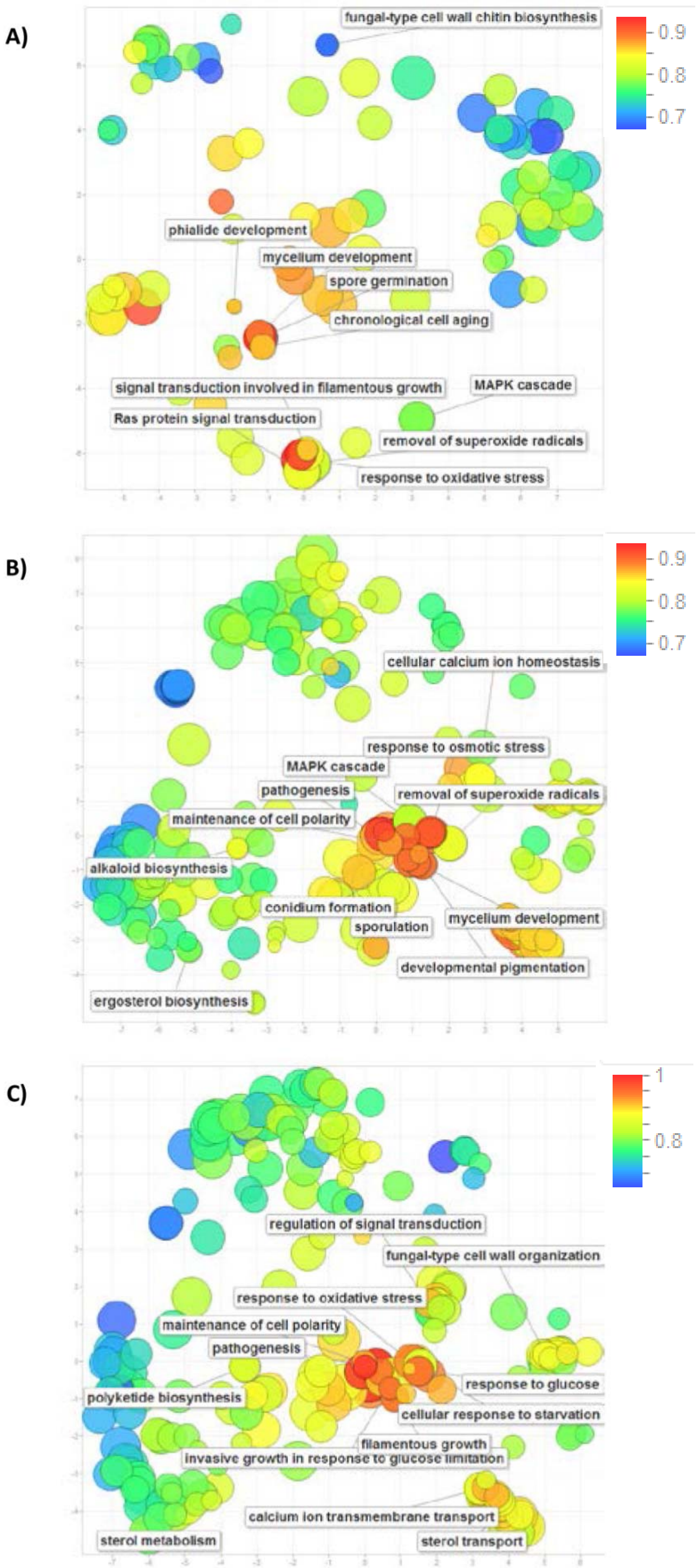

Additional file 5. REVIGO scatterplots of biological process GO terms associated with *Cp*20.1 genes of high (A), medium (B) and low (C) *in planta* expression. Each circle represents a GO term. Semantically similar GO terms lie close together. Their color represents uniqueness within the GO terms. Their size indicates the frequency in the underlying GO annotation database (used: UniProt). As the number of GO terms processed by REVIGO is limited, terms associated with high, medium and low expressed genes and some general terms in low expressed genes had to be omitted as data input. Labelled are terms which are characteristic for a fungus like *C. purpurea*.
