# Supplementary material for: Cross-talk of the biotrophic pathogen Claviceps purpurea and its host Secale cereale
Source: BMC Genomics. 2017 Apr 4;18:273. doi: 10.1186/s12864-017-3619-4 (PMC5379732; doi:10.1186/s12864-017-3619-4)
Supplement: Supplementary file 9 — Microscopical analyses of strain Cp20.1 mCherry:NLS (PDF 491 kb) [file 12864_2017_3619_MOESM9_ESM.pdf]

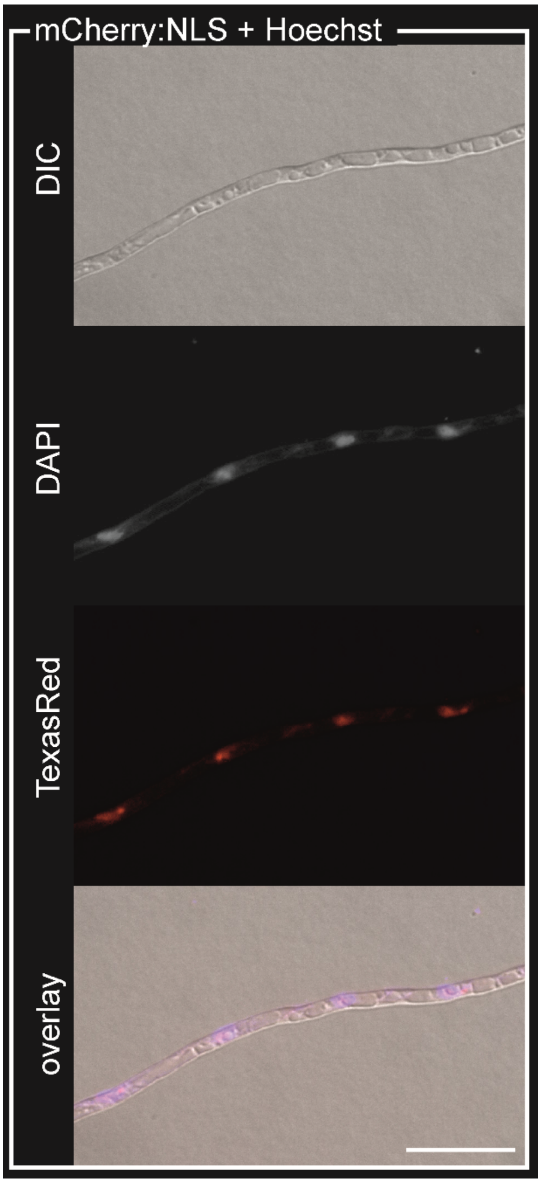

Additional file 9. Microscopical analyses of strain *Cp*20.1 mCherry:NLS (under control of the constitutive oliC-promoter). The strain was cultivated on medium covered objective slides and incubated for 3 days. Strong fluorescence could be observed within fungal nuclei. Localization was controlled by using Hoechst staining. Top to bottom: DIC; DAPI; mCherry (TexasRed), overlay (Bars =20µm).
