# Supplementary material for: Cross-talk of the biotrophic pathogen Claviceps purpurea and its host Secale cereale
Source: BMC Genomics. 2017 Apr 4;18:273. doi: 10.1186/s12864-017-3619-4 (PMC5379732; doi:10.1186/s12864-017-3619-4)
Supplement: Supplementary file 10 — Deletion strategy of cp1105 and cp8623 and identification of deletion strains (PDF 281 kb) [file 12864_2017_3619_MOESM10_ESM.pdf]

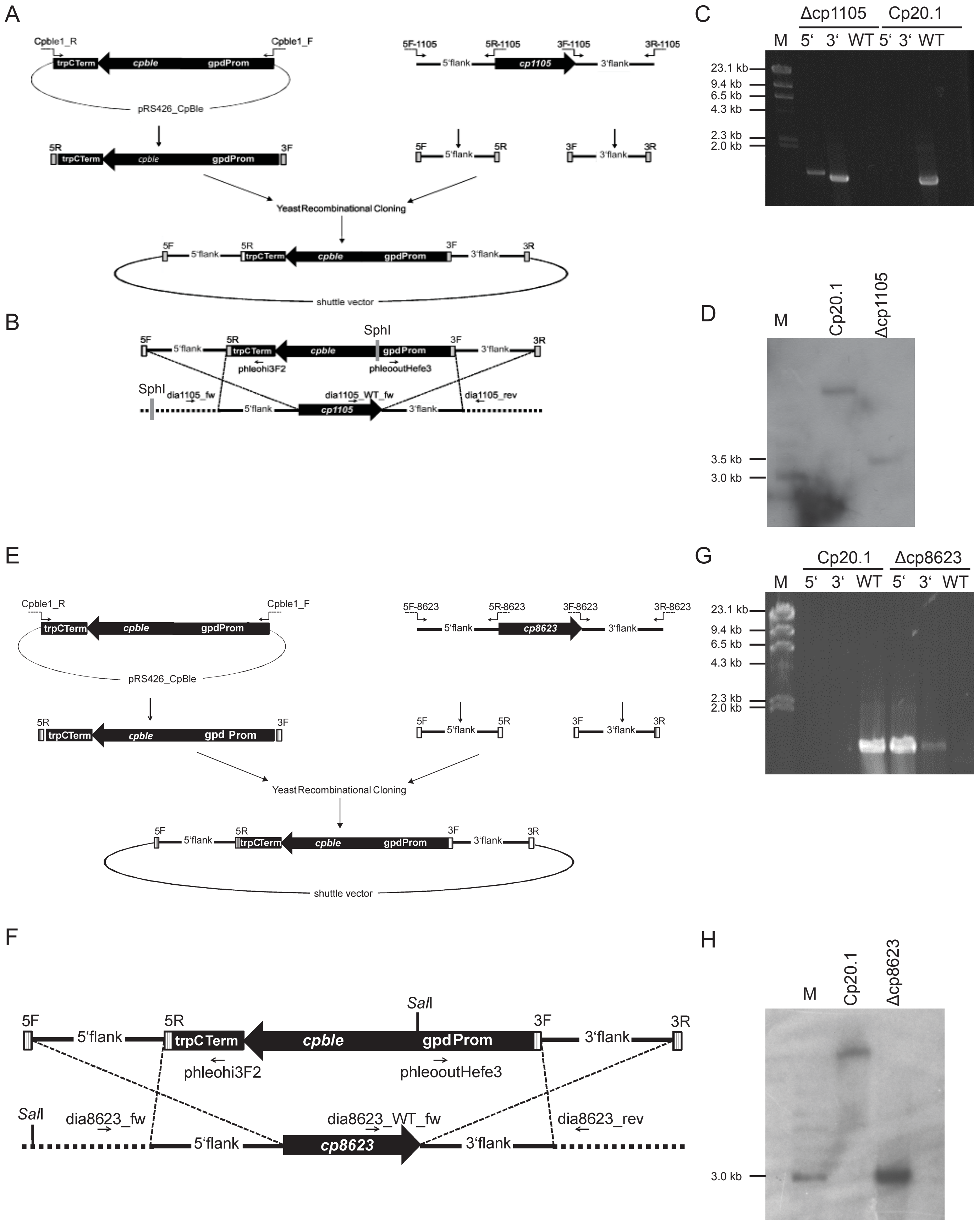

Additional file 10: Generation of the Δcp1105 (A-D) and Δcp8623 mutant (E-H). A/E) The replacement vector was obtained by the yeast recombinational method. It was constructed by cloning the 3‘and 5‘flanking regions of cp1105/cp8623 on each side of the phleomycin resistance cassette into the yeast shuttle vector pRS426 plasmid (see materials and methods for further details). The resulting replacement fragment was used to transform C. purpurea wild type strain 20.1. B/F) The mutant was generated by homologous integration of the resistance cassette via a double cross over event between the homologous regions of the replacement fragment and the genomic region of cp1105/cp8623. Primers used for diagnostic PCRs are indicated. Primers and destination vectors are not drawn to scale. C/G) Diagnostic PCRs of Δcp1105/Δcp8623 and Cp20.1. For the deletion mutant a homologous integration event is documented by amplification of 5´and 3´ diagnostic fragments, resp., while lack of the wild type control fragments proves the absence of the wt gene in the mutant (λ restricted with HindIII, fragment sizes are indicated on the left). D) Southern Blot analyses of Δcp1105. SphI digested genomic DNA of Cp20.1 and Δcp1105 was probed with the 5'-flank of cp1105. Lack of wt fragment and single integration of the replacement fragment is evident. H) Southern Blot analyses of Δcp8623. SalI digested genomic DNA of Cp20.1 and Δcp8623 was probed with the 5‘-flank of cp8623. Lack of wt fragment and single integration of the replacement fragment is evident.
