# Supplementary material for: Cross-talk of the biotrophic pathogen Claviceps purpurea and its host Secale cereale
Source: BMC Genomics. 2017 Apr 4;18:273. doi: 10.1186/s12864-017-3619-4 (PMC5379732; doi:10.1186/s12864-017-3619-4)
Supplement: Supplementary file 11 — Deletion strategy of cp3095/cp3096 and cp5492/cp5493 and identification of deletion strains (PDF 209 kb) [file 12864_2017_3619_MOESM11_ESM.pdf]

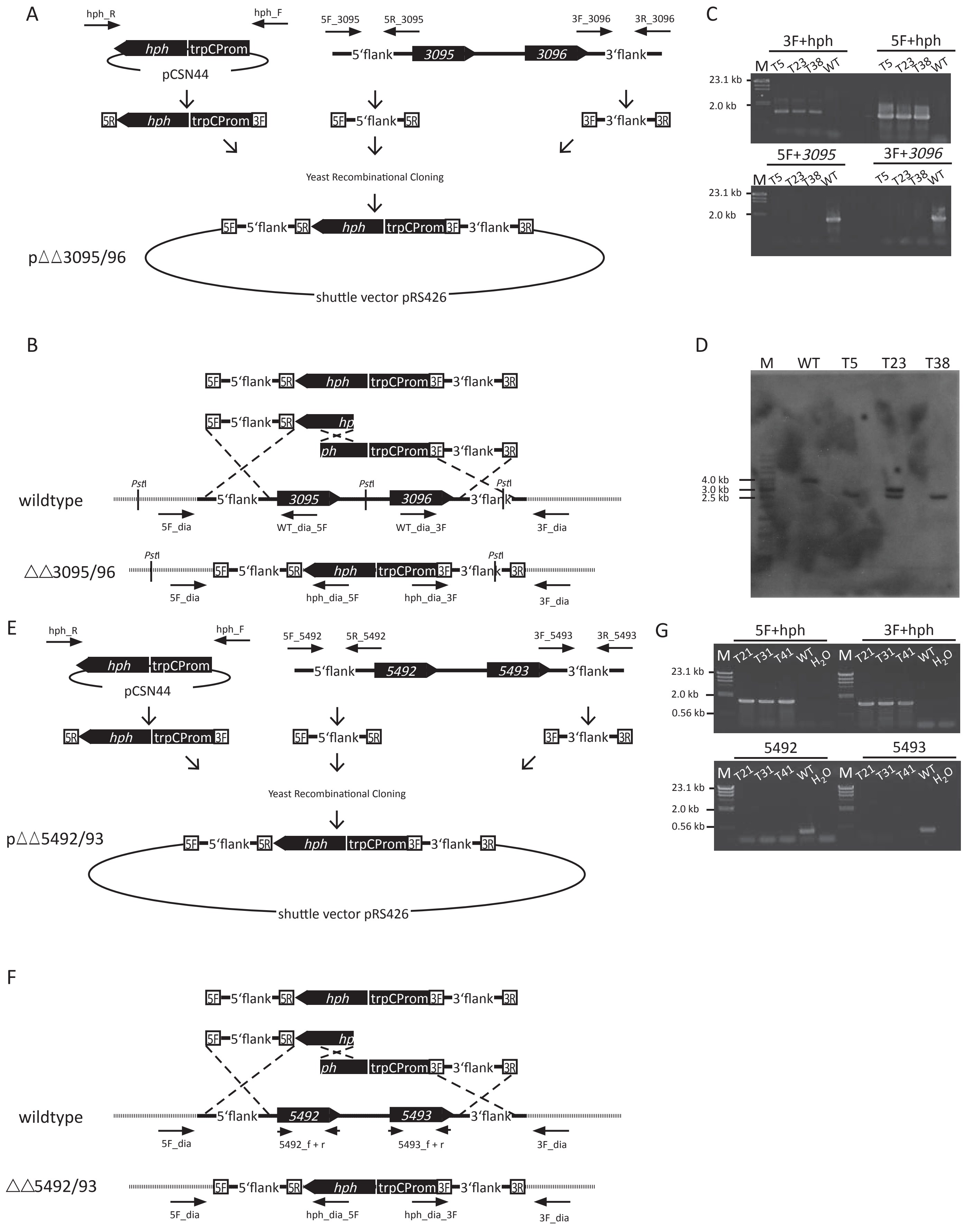

Additional file 11: Generation of the Δcp3095/96 (A-D) and Δcp5492/93 mutants (E-H). A/E) The replacement vector was obtained by the yeast recombinational method. It was constructed by cloning the 3'and 5'flanking regions of cp3095/96/cp5492/93 on each side of the hygromycin resistance cassette into the yeast shuttle vector pRS426 plasmid (see materials and methods for further details). The resulting replacement fragment was used to transform *C. purpurea* wild type strain 20.1. B/F) The mutant was generated by homologous integration of the resistance cassette via a triple cross over event between the homologous regions of the replacement fragment and the genomic region of cp3095/96/cp5492/93 and the overlaps of the hygromycin gene. Primers used for diagnostic PCRs are indicated. Primers and destination vectors are not drawn to scale. C/G) Diagnostic PCRs of Δcp3095/96/Δcp5492/93 and Cp20.1. For the deletion mutants a homologous integration event is documented by amplification of 5´and 3´ diagnostic fragments, resp., while lack of the wild type control fragments proves the absence of the wt gene in the mutants (λ restricted with HindIII, fragment sizes are indicated on the left). D) Southern Blot analyses of Δcp3095/96. PstI digested genomic DNA of Cp20.1 and Δcp3095/96 T5, T23, T38 was probed with the 5'-flank of cp3095/96. Lack of wt fragment and single integration of the replacement fragment is evident for T5 and T38
