# Supplementary material for: Cross-talk of the biotrophic pathogen Claviceps purpurea and its host Secale cereale
Source: BMC Genomics. 2017 Apr 4;18:273. doi: 10.1186/s12864-017-3619-4 (PMC5379732; doi:10.1186/s12864-017-3619-4)
Supplement: Supplementary file 12 — Growth assay of Δ1105 and Δ8623 (PDF 43 kb) [file 12864_2017_3619_MOESM12_ESM.pdf]

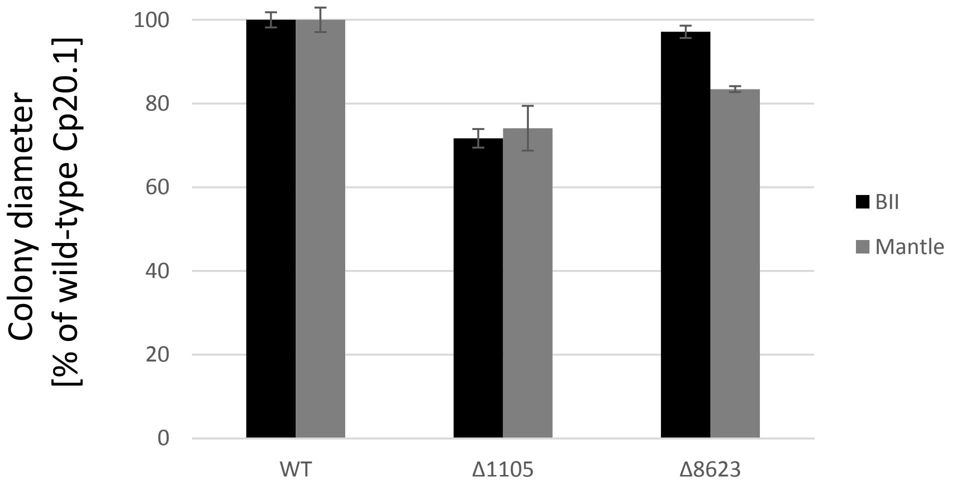

Additional file 12. Growth assay of single effector deletion strains. The strains were cultivated on BII (complete) and Mantle (sporulation) medium. The bars represent average values of at least four colonies measured 12 dpi.
