# Supplementary material for: Cross-talk of the biotrophic pathogen Claviceps purpurea and its host Secale cereale
Source: BMC Genomics. 2017 Apr 4;18:273. doi: 10.1186/s12864-017-3619-4 (PMC5379732; doi:10.1186/s12864-017-3619-4)
Supplement: Supplementary file 13 — Oligonucleotides used in this study (PDF 82 kb) [file 12864_2017_3619_MOESM13_ESM.pdf]

Additional file 13: Oligonucleotides used in this study

Name (this stuy)	intern (if differing)	Sequence	
**Primer used for Generation of deletion constructs**			
hph_F	hphF-trpC-P (hphF)	gacagaagatgatattgaaggagc	
hph_R	hphF-trpC-P (hphR)	gatttcagtaacgttaagtggat	
CpBle1F		CGGAGACAGAAGATGATATTGAAGGAGCGATCGAGACCTAATACAGCCCC	
CpBle1R		GTTGGAGATTTCAGTAACGTTAAGTGGGCATTGCAGATGAGCTGTATCTG	
5R_3095	YRC_3095_5F_ble_rev	ACTTAACGTTACTGAAATCTCCAACTATAGACCTACCTTGAGAGAACCC	
3F_3096	YRC_ble_3096_3F_fw	TTCAATATCATCTTCTGTCTCCGACTACCTGCATAGAACGAACTACG	
3R_3096	YRC_3096_3F_pRS_rev _neu	caatttcacacaggaaacagcCCAAGCTTTTGCGATGGCTTCCCTCTTC	
5F_3095	YRC_pRS_3095_5F_fw _neu	CCAGGGTTTTCCCAGTCACGACgaagcttGGCTCTACAGGCTTTGCATC	
3F_5493		CTCCTTCAATATCATCTTCTGTCTCCGACGCGAAAGGAAAGAAATGGT	
5F_5492	5F_5492_neu	GGTTTTCCCAGTCACGACGctcgagGTGTATGTACAGAGTACATATGTAC	
3R_5493	3R_5493_neu	acaatttcacacaggaaacagcctcgagAGAGGACAGCGTCTGATCC	
5R_5492		ACTTAACGTTACTGAAATCTCCAACGTTGTATGTTTGATGTGATATTGG	
5F_1105		AGGGTTTTCCCAGTCACGACGGAATTCTATCTACAACGGACACTCGC	
5R_1105		CCACTTAACGTTACTGAAATCTCCAACGGTGGCGGTTTAGGGAAAG	
3F_1105		ATATCATCTTCTGTCTCCGACGCGTTCATTGTCCAACGATGG	
3R_1105		AACAATTTCACACAGGAAACAGCGAATTCTTAGATTCCGCTCTGTCGG	
5F_8623		AGGGTTTTCCCAGTCACGACGGAATTCTAAGTTGCTCAATTCCGTTGC	
5R_8623		CCACTTAACGTTACTGAAATCTCCAACGGTCACTAGTTTTGGTTAT	
3F_8623		TTCAATATCATCTTCTGTCTCCGACGAAGTCATTGCTCATCATG	
3R_8623		CAATTTCACACAGGAAACAGCGAATTCAGACTCATACCTAGACGTCC	
tripleNLS_fw_2		GCGGCCGCAAAGTCCGGACTCAGATC	
tripleNLS_rev		TGCGCGGCCGCTATACCTTTCTCTT	
**Primer used for Generation of mCherry and NLS fusion constructs**			
Cp3095_mCh_rev	YRC_3095_mCh_rev	ttacCTCGCCCTTGCTTACCATGTCGCAGTTGCCTACAGCG	
Cp3095_P_fw	YRC_ptrpC_nat3095_fw	AAATGCTCCTTCAATATCATCACCAGGGTGTATTGCAGCG	
Cp5492_P_fw	YRC_ptrpC_nat5492_fw	CAAAAAATGCTCCTTCAATATCTCCTAGCTTCACGGACCACGTGAGC	
Cp5492_mCh_rev	YRC_5492_mCherry_rev	cttacCTCGCCCTTGCTTACCATAGGGGCGCACCAGACAGTGC	
Cp1105_P_fw		CCCAAAAAAATGCTCCTTCAATATCTATCTACAACGGACACTCGC	
Cp1105_mCherry_rev		TACTTACCTCGCCCTTGCTTACCATTTTGCCGTTGCATCTGTTTC	
Cp5493_P_fw		GCCCAAAAAAATGCTCCTTCAATATCAGTTGACAGTTTGGCGGGATG	
Cp5493_mCherry_rev		TACTTACCTCGCCCTTGCTTACCATAGCGCAGTAGATCATACCAAT	
Cp7156_P_fw		GCCCAAAAAAATGCTCCTTCAATATCTACCTGCCACTTGCTGGCG	
Cp7156_mCherry_rev		TACTTACCTCGCCCTTGCTTACCATCAGCTCATCATTAGTTTGCT	
Cp8623_P_fw		AAAAAATGCTCCTTCAATATCTAAGTTGCTCAATTCCGTTGC	
Cp8623_mCherry_rev		TACTTACCTCGCCCTTGCTTACCATGTCATACAAATGGTGAATAC	
**Primer used for Diagnostic PCRs**			
Phleohi3F2		gtgttcaggatctcgataagatacg	
phleo_out Hefe 3		gagctcggtataagctctcc	
hph_dia_3F	pCSN44-trpC-P2	GTGATCCGCCTGGACGACTAAACC	
hph_dia_5F	Hph-hiF	GTCTGGACCGATGGCTGTGTAGAAG	
5493_r	5493rev	cgcttaagcgcagtagatc	
5492_f	5492fw	atgaaagtcattgctgcc	
5492_r	5492rev	caagttttcgcttaaggggc	
5493_f	5493fw	atgcagctcatttccgttc	
3F_dia_3096	dia_3096_3F_rev	TGTCCTCATGCCTGGAACTCG	
5F_dia_3096	dia_3095_5F_fw	AACCATGGACAAGAACTACTGG	
WT_dia_5F_3095	3095_gDNA_rev	CCTGACTCCTTCTCAGTCTTCCC	
WT_dia_3F_3096	RT_3096_fw	TTGCATCTTGATTGGTGTGCGGC	
dia_Cp1105_fw		CACTTGCAATCACAATCAAGCC	
dia_Cp1105_rev		AGGAGACATCTGTGATACTAGACTG	
dia_Cp1105_WT_fw		GGTCGAGTGTATTTCCCAGAT	
dia_Cp8623_fw		TCATTCTGCAATGCACGAAGG	
dia_Cp8623_rev		AGAGGTTCAGTTGATCGCATTCGG	
dia_Cp8623_WT_fw		AGCGCACATAACCATCTACG	
**Primer used for qRT-PCR**			**Gene**
Actin uni		GCCGTTTTCCCCTCTATCGTC	CPUR_01270.1
Actin rev		ACATACGAGTCCTTCTGACCCAT	CPUR_01270.1
Gpd uni		CCCGAATATGCTGCCTACATGCT	CPUR_00276.1
Gpd rev		CGTCCTTCTTGATCTCGCCCT	CPUR_00276.1
Tub uni		TACAATGGTACCTCGGAGCAAC	CPUR_08361.1
Tub rev		CCAGAGGCCTCATTGAAGTAGAC	CPUR_08361.1
Pls1_F1	Cp_Pls1_F1	ATGGGACGCACCGCCAATAT	cpur_00527
Pls1_R1	Cp_Pls1_R1	CTAAAACGTGCGAAAACCAC	cpur_00527
RT_xyl1_fw		TCTCGGCACGAACACGTTCC	cpur_03570
RT_xyl1_rev		ATATGTCCCGAGCGGCATGC	cpur_03570
RT_5492_fw		ACCAGGTTACAAGGCCTACCC	cpur_05492
RT_5492_rev		TTCTCACAGCCAGTGCCTCC	cpur_05492
RT2_3095_rev		AACAGACAAATGTTCCCTTTTCTGTGC	cpur_03095
RT2_3095_fw		ATTGGCAGACAAATTGCATCATCGC	cpur_03095
RT_3096_rev		AGCATTCAACAGACACTGTTCCCTTTGC	cpur_03096
RT_3096_fw		TTGCATCTTGATTGGTGTGCGGC	cpur_03096
RT_1105_fw		CGATGGATCGCACTGCTGCGTC	cpur_01105
RT_1105_rev		GAGAGTTGCCAGTGATCTGGG	cpur_01105
RT_8623_fw2		GCTGGCATTGCTGTCGCCAAG	cpur_08623

RT_8623_rev2		TCCGAAGTCTGCAGCGATCGAGT	cpur_08623
RT_7156_fw		TCTGAAGATGCTGCTGTCCATGTGG	cpur_07156
RT_7156_rev		ATGCGCTTCGCCATGATGAACG	cpur_07156
dia_Cp5493_WT_fw		TGTGACCACGGAACACATGG	cpur_05493
RT_5493_rev		ACACGCAGTTGCTATCACCATCC	cpur_05493
ADP_fw	ScADP_RF_a_fw	TCTCATGGTTGGTCTCGATG	Ta2291*
ADP_rev	ScADP_RF_a_rev	GGATGGTGGTGACGATCTCT	Ta2291*
RLI_fw	ScRLI_a_fw	TCGAGCAACTCATGGACCAA	Ta2776*
RLI_rev	ScRLI_a_rev	GCTTTCCAAGGCACAAACAT	Ta2776*
CDC_fw	ScCDC_a_fw	CAGCTGCTGACTGAGATGGA	Ta54227*
CDC_rev	ScCDC_a_rev	ATGTCTGGCCTGTTGGTAGC	Ta54227*
RT-Rye_1_fw		GAACGAGGAGATGAAGCTCG	Sc1Loc01149658.2
RT-Rye_1_rev		GGCAGGTCTCCACAAACATT	Sc1Loc01149658.2
RT_Rye_3_fw2		GGAGTACGAGCTGGTGATCA	Sc2Loc00096015.6
RT_Rye_3_rev2		GTCTTGTCCGACTCGATGGA	Sc2Loc00096015.6
RT-Rye_4_fw		ATGGAGGTGTACCTGTTCGG	Sc5Loc00240479.1
RT-Rye_4_rev		AAGGTGCAGGCGTAGTTCAC	Sc5Loc00240479.1
RT-Rye_5_fw		GAGGCAATGAAGCAGGACAT	Sc2Loc02172093.1
RT-Rye_5_rev		GCTGACGAAGAGGTGGTAGC	Sc2Loc02172093.1
RT-Rye_6_fw		AGGACCTCCCACACCTCTCT	Sc3Loc01905034.2
RT-Rye_6_rev		TTGTTGCATATCCTCACCCA	Sc3Loc01905034.2
RT_rye8_fw		CGAGCGACAAGGAGTGCAAC	Sc4Loc00580338.2
RT_rye8_rev		TGTCCTTCTTCTGCGCCACC	Sc4Loc00580338.2

*wheat unigene (GenBank database)
